# The persistence and oscillations of submicroscopic *Plasmodium falciparum* and *Plasmodium vivax* infections over time in Vietnam: an open cohort study

**DOI:** 10.1016/S1473-3099(18)30046-X

**Published:** 2018-05

**Authors:** Thuy-Nhien Nguyen, Lorenz von Seidlein, Tuong-Vy Nguyen, Phuc-Nhi Truong, Son Do Hung, Huong-Thu Pham, Tam-Uyen Nguyen, Thanh Dong Le, Van Hue Dao, Mavuto Mukaka, Nicholas PJ Day, Nicholas J White, Arjen M Dondorp, Guy E Thwaites, Tran Tinh Hien

**Affiliations:** a Oxford University Clinical Research Unit, Wellcome Trust Major Oversea Programme, Ho Chi Minh City, Vietnam; b Mahidol Oxford Tropical Medicine Research Unit, Faculty of Tropical Medicine, Mahidol University, Bangkok, Thailand; c Centre for Tropical Medicine and Global Health, Nuffield Department of Medicine, Churchill Hospital, Oxford, UK; d Institute of Malariology, Parasitology, and Entomology (IMPE), Ho Chi Minh City, Vietnam; e Center for Malariology, Parasitology and Entomology Control, Ninh Thuan Province, Vietnam

## Abstract

**Background:**

A substantial proportion of *Plasmodium* species infections are asymptomatic with densities too low to be detectable with standard diagnostic techniques. The importance of such asymptomatic plasmodium infections in malaria transmission is probably related to their duration and density. To explore the duration of asymptomatic plasmodium infections and changes in parasite densities over time, a cohort of participants who were infected with *Plasmodium* parasites was observed over a 2-year follow-up period.

**Methods:**

In this open cohort study, inhabitants of four villages in Vietnam were invited to participate in baseline and subsequent 3-monthly surveys up to 24 months, which included the collection of venous blood samples. Samples were batch-screened using ultra-sensitive (u)PCR (lower limit of detection of 22 parasites per mL). Participants found to be infected by uPCR during any of these surveys were invited to join a prospective cohort and provide monthly blood samples. We estimated the persistence of *Plasmodium falciparum* and *Plasmodium vivax* infections and changes in parasite densities over a study period of 24 months.

**Findings:**

Between Dec 1, 2013, and Jan 8, 2016, 356 villagers participated in between one and 22 surveys. These study participants underwent 4248 uPCR evaluations (11·9 tests per participant). 1874 (32%) of 4248 uPCR tests indicated a plasmodium infection; 679 (36%) of 1874 tests were *P falciparum* monoinfections, 507 (27%) were *P vivax* monoinfections, 463 (25%) were co-infections with *P falciparum* and *P vivax*, and 225 (12%) were indeterminate species of *Plasmodium*. The median duration of *P falciparum* infection was 2 months (IQR 1–3); after accounting for censoring, participants had a 20% chance of having parasitaemia for 4 months or longer. The median duration of *P vivax* infection was 6 months (3–9), and participants had a 59% chance of having parasitaemia for 4 months or longer. The parasite densities of persistent infections oscillated; following ultralow-density infections, high-density infections developed frequently.

**Interpretation:**

Persistent largely asymptomatic *P vivax* and *P falciparum* infections are common in this area of low seasonal malaria transmission. Infections with low-density parasitaemias can develop into much higher density infections at a later time, which are likely to sustain malaria endemicity.

**Funding:**

The Wellcome Trust, Bill & Melinda Gates Foundation.

## Introduction

In the past 20 years, it has become apparent that in low-transmission settings such as southeast Asia many *Plasmodium* spp infections remain asymptomatic, and most of these cannot be detected by light microscopy because of low parasite densities.^1^, ^2^, ^3^ Estimating the submicroscopic plasmodium reservoir has become more accurate with the development of a high blood volume (1 mL), ultrasensitive, quantitative PCR (uPCR) method with a diagnostic sensitivity as low as 22 parasites per mL, allowing the detection of more than 85% of all *Plasmodium vivax* parasitaemias and more than 70% of all *Plasmodium falciparum* parasitaemias.^4^

Persistent, asymptomatic plasmodium infections carry health risks for the infected individual including chronic anaemia, increased risks of maternal and neonatal mortality, impaired immune competence resulting in co-infections with invasive bacterial diseases, and cognitive impairment.^5^ At the public health level, asymptomatic, submicroscopic infections provide a parasite reservoir for malaria transmission. In areas with low and seasonal transmission, persistent asymptomatic parasite carriage bridges the dry season, during which mosquito numbers are very low and malaria transmission is almost absent, thus sustaining endemicity. Most low-density plasmodium infections detected by uPCR also have low gametocyte densities, which could be insufficient to transmit the infection to mosquito vectors at that time because the mosquito has to ingest at least one male and one female gametocyte in a blood meal to propagate the infection.^6^ This observation has led some researchers to question the public health importance of low-density parasite infections. However, studies concluding that infections with low gametocyte densities are not transmissible assume that parasite densities are static. If persistence of plasmodium infection is associated with oscillations of asexual and consequently sexual parasite densities to transmissible levels, submicroscopic parasitaemia is important by maintaining infections in a community and triggering seasonal malaria outbreaks.

**Research in context**

**Evidence before this study**

We searched PubMed and Google Scholar for studies on persistence and oscillations of *Plasmodium* species infections up to Oct 29, 2017. We searched PubMed using the MeSH terms “plasmodium”, and the (all fields) terms “persistence” or “duration” and “oscillation” and “dynamics” restricted to cohort studies or trials. 143 papers included in the search terms of which 14 addressed the persistence and oscillations in parasite densities. In high-transmission settings, repeated malaria infections induce partial immune responses in the human host, rendering most plasmodium infections asymptomatic in older children and adults. In low-transmission settings such as southeast Asia, the proportion of asymptomatic carriers is much larger than previously appreciated. Although substantial evidence from high-transmission settings suggests that persistence of untreated asymptomatic *Plasmodium falciparum* infections is an important source of transmission, this is not known for low-transmission settings.

**Added value of this study**

The limitations of contemporary cohort studies on the persistence of plasmodium carriage include the challenges of frequent sampling over longer periods, as well as the limited sensitivity of conventional diagnostic tools (light microscopy, rapid diagnostic tests, and PCR based on dried blood samples) to detect very low-density parasitaemias. We used an ultrasensitive quantitative PCR method, which has a much lower limit of detection. Our study reports on a cohort of asymptomatic participants infected with *P falciparum, Plasmodium vivax*, or both, who were followed up for 24 months and documents the frequent persistence of infections over extended periods. The study shows large fluctuation in parasite densities during a single episode, oscillating by orders of magnitude over time, with a substantial proportion of participants developing symptomatic disease.

**Implications of all the available evidence**

The submicroscopic, asymptomatic *Plasmodium* spp reservoir could have a crucial role in sustaining malaria transmission in low and seasonal transmission settings and can explain the seasonal malaria increase after the dry season. Screening interventions that rely on standard diagnostic tools such as rapid diagnostic tests will not identify a large proportion of the asymptomatic low-density plasmodium reservoir. Targeting the entire asymptomatic plasmodium reservoir with antimalarial treatment is important to accelerate malaria elimination.

Here, we explore the persistence of plasmodium infection parasitaemia and density levels over a 2-year study period in a large cohort of asymptomatic malaria-infected participants in Vietnam.

## Methods

### Study design and participants

This was an open cohort study nested in a village randomised trial in Vietnam to evaluate the effect of mass drug administrations on the prevalence of *P falciparum* infections. Villagers found to be infected with *P falciparum, P vivax,* mixed *P falciparum* and *P vivax*, or unidentified *Plasmodium* spp during baseline mass drug administration or follow-up surveys were invited to join the study.

Mass drug administrations were done in two villages in Dak O commune, Binh Phuoc province (Bu Bung, Bu Khon) and two villages in Phuoc Ha Commune, Ninh Thuan province (Gia, Tan Ha) in Vietnam. In 2015, the annual *P falciparum* malaria incidence in Dak O commune was 25 cases in 1000 people and the *P vivax* malaria incidence was 17 cases in 1000 people.^7^ In Phuoc Ha Commune, the annual *P falciparum* malaria incidence was 24 cases in 1000 people and *P vivax* malaria incidence was seven cases in 1000 people.^7^ The villages were selected based on parasite prevalence, enthusiasm of villagers to participate, and access (road conditions). Randomisation was used to determine which villages in each province received the mass drug administration in the first year (baseline) and in the second year (month 12) of the study.

Ethical approval for the study was received from the Institute of Malariology, Parasitology and Entomology in Ho Chi Minh City (185/HDDD), the Institute of Malariology, Parasitology and Entomology in Qui Nhon, and the Oxford Tropical Research Ethics Committee (1015-13). Individual written informed consent was obtained from those aged 16 years and older and parental or guardian consent was obtained for children younger than 16 years.

### Procedures

The mass drug administrations were done with the aim to accelerate the elimination of *P falciparum* malaria from study villages. They were embedded in several malaria elimination strategies including community engagement, improved case management, and vector control strategies. The drug regimen consisted of three monthly rounds (baseline, month 1, and month 2) of three once per day treatment doses of oral dihydroartemisinin [DHA] and piperaquine (7 mg/kg dihydroartemisinin and 55 mg/kg piperaquine phosphate) combined with a single low dose of oral primaquine (15 mg or 0·25 mg/kg). Frequency and timing of the mass drug administration rounds was based on the modelled maximum effects in transmission reduction and the post-treatment prophylactic effect of piperaquine, which is around 30 days for sensitive strains.^8^, ^9^ All residents in the study villages were encouraged to take part in three rounds of drug administration except for women in the first trimester of pregnancy and children younger than 6 months. A single low dose of primaquine is sufficient to rapidly clear gametocytes, which are not susceptible to schizonticidal drugs, but does not clear hypnozoites and therefore does not prevent *P vivax* relapse.^10^ During mass drug administration, all drugs were administered under direct observation of study staff. The effect of the mass drug administration on the prevalence of plasmodium will be reported separately.

**Figure 1:**
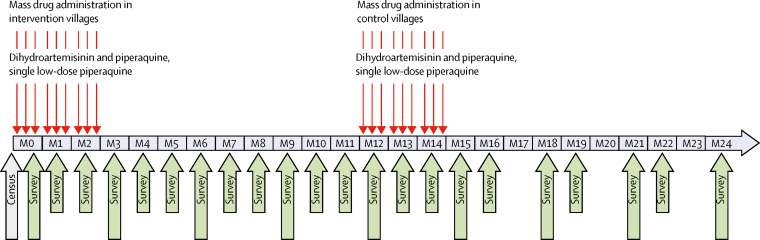
Study design Thin red arrows indicate the days of mass drug administrations. Large grey arrow indicates date of study population count (census). Large green arrows indicate the timing of surveys in which all residents were invited. Small green arrows indicate the timing of surveys for cohort members only. Surveys at months 17, 20, and 23 were cancelled because of logistical reasons. M=month.

The surveillance period was 24 months; directly preceding the mass drug administration and then in intervals of 3 months (0, 3, 6, 9, 12, 15, 18, 21, and 24 months; figure 1). All people residing in the study villages and who were 6 months or older, including temporary residents and migrant workers, were invited to participate in cross-sectional surveys. We collected demographic information and measured the tympanic temperature, weight, and height of all individuals. We collected 3 mL venous blood from all individuals aged 5 years or older, and 500 μL from children aged 6 months to 5 years. Participants with temperature of 37·5°C or higher (fever) were tested for malaria by a rapid diagnostic test (RDT; SD Bioline Malaria Ag Pf/Pv, Gyeonggi-do, South Korea), and were treated according to national guidelines if positive by RDT or thought to have malaria based on clinical presentation. We invited individuals found to be infected with plasmodium during the quarterly surveys by uPCR to provide monthly blood samples until the completion of the 24-month study. The surveys at months 17, 20, and 23 were cancelled due to logistical reasons. Cohort members could participate in up to 22 surveys.

Blood samples were stored in cooling boxes in the field and then transported within 6 h to the local laboratory for further sample processing. Sample processing for the quantitative uPCR included separation of plasma, buffy coat, and packed red blood cells, which were frozen and stored at −80°C. The frozen samples were shipped to Ho Chi Minh City, Vietnam for DNA extraction and quantitative uPCR. Detailed description, evaluation, and validation of the high-volume ultrasensitive quantitative uPCR methods have been reported previously.^11^ Briefly, purified DNA was dehydrated in a centrifugal vacuum concentrator and then suspended in a small volume of PCR-grade water. We assessed the presence of malaria parasites and an estimate of the parasite numbers (assessed as genomes) in each sample by an absolute quantitative real-time PCR (rtPCR) method. 18S rRNA-targeting primers and hydrolysis probes used in the assay have been validated and are highly specific for *Plasmodium* species.^12^ The lower limit of accurate quantitation of this method is 22 parasites per mL of whole blood.^2^, ^4^ We analysed uPCR-positive samples using nested PCR protocols specific to *P falciparum* (microsatellite marker Pk2), *P vivax* (microsatellite marker 3.502), and *Plasmodium malariae* (18s rRNA).^12^ Samples with insufficient DNA or where no amplification was obtained in this step were reported as being of indeterminate species (*Plasmodium* spp). We defined a high-density infection based on observed distributions as any infection with more than 200 000 parasites per mL.

### Outcomes

An infection with one *Plasmodium* species can transit between a monoinfection, a mixed species infection, and an infection in which the *Plasmodium* species cannot be identified. To follow the progress of infections over time, we grouped *P falciparum* monoinfections with mixed infections (*P falciparum* and *P vivax*) and unidentified species occurring before or after a *P falciparum* infection. *P vivax* monoinfections were grouped with *P falciparum* and *P vivax* mixed infections and unidentified species. The use of group definitions results in an overlap between the *P falciparum* and the *P vivax* group infections (appendix). If an episode of infection had a missing value at the next timepoint for two consecutive points after the initial plasmodium infection, the episode was censored and a 1-month duration recorded (eg, positive, missing, missing would be recorded as 1 month). If an episode had a missing value after the initial plasmodium infection, and was then positive at the next timepoint, it was regarded as a persistent positive from the initial point (eg, positive, missing, positive, positive, positive, positive would be recorded as 6 months). If an episode was transiting between a missing and a positive value for several consecutive months, then the episode was assumed positive throughout (eg, positive, missing, positive, missing, positive, missing, positive, missing, positive, missing would be recorded as 10 months). If a *P falciparum* group episode had treatment with DHA and piperaquine at the next timepoint, the episode was censored at the treatment time. In the absence of a radical cure with primaquine, it was assumed that a *P vivax* infection persisted. Infections present before and after the missing months 17, 20, and 23 were assumed to be present during the missing month.

### Statistical analysis

Based on the above definitions, episodes of *P falciparum* or *P vivax* were identified in the database and duration of each episode was calculated in months. For these data, time to clearance of plasmodium was analysed using survival methods. We calculated Kaplan-Meier estimates and superimposed 95% Greenwood CIs on the plots. We took into account censoring in the Kaplan Meier plots. We assessed the relationship between parasitaemia and time since the episode began and used linear regression modelling to explore linear relationships between time since the episode started and parasitaemia. The slope of the regression line, 95% CIs, and p values were calculated. Parasite density data were log-normalised for statistical analysis. We used Spearman's rank correlation coefficients to assess correlations between continuous variables. The exponential model of the form:

$$y=e^{\beta X}$$

were fitted to assess relationship between parasite densities (on a natural log scale) and duration, where y is duration of infection and x is log of parasite density. This model was used for *P falciparum* and *P vivax*. We used Kruskal-Wallis or *t* tests to assess the correlation between continuous and categorical variables depending on the number of groups, and a Wilcoxon-type test for trend to assess the statistical significance of trends.^13^ We accounted for the fact that the same individuals contributed to several episodes by using robust standard errors. All tests were done at the 95% significance level. All analyses were done in Excel (version 14) and Stata (version 14.2).

### Role of the funding source

The funders of this study had no role in study design, data collection, analysis, or interpretation, or writing of the report. The corresponding author had full access to all the data in the study and had final responsibility for the decision to submit for publication.

## Results

Between Dec 1, 2013, and Jan 8, 2016, 356 participants were recruited for this study. The cohort included 356 participants with parasitaemia; the median age was 35 years (IQR 25·3–45·3) and 238 (67%) participants were male (table). The cohort members participated in between one and 22 surveys (appendix). The surveillance period started in Dec 1, 2013, and ended 25 months later. 356 cohort members had 4248 uPCR tests, 11·9 tests per member. Overall, 1874 (32%) of 4248 uPCR tests detected a *Plasmodium* spp infection. 679 (36%) of 1874 were *P falciparum* infections, 507 (27%) were *P vivax* infections, and 463 (25%) of 1874 were co-infected with *P falciparum* and *P vivax*. The species of the remaining 225 (12%) of 1874 could not be identified.

**Table:** Baseline characteristics of participants and non-participants by village

		**Bu Bung**		**Bu Khon**		**Gia**		**Tan Ha**		**Total**	
		N	Median (IQR) or n (%)	N	Median (IQR) or n (%)	N	Median (IQR) or n (%)	N	Median (IQR) or n (%)	N	Median (IQR) or n (%)
**Participants**											
Age (years)		93	33·3 (24.3–44·3)	80	37·8 (24·8–45·3)	119	34·3 (25·3–51·3)	62	36·3 (25·3–44·3)	354	35·3 (25·3–45·3)
Weight (kg)		66	50·8 (44·0–57·0)	55	52·5 (43·5–57·0)	93	46·0 (39·0–51·0)	53	47·0 (40·0–51·0)	267	49·0 (40·5–54·0)
Height (cm)		64	160 (151–165)	55	157 (147–165)	93	149 (140–156)	53	148 (144–156)	265	153·0 (145·0–160·0)
Sex											
	Male	93	68 (73%)	81	65 (80%)	120	69 (58%)	62	36 (58%)	356	238 (67%)
	Female	93	25 (27%)	81	16 (20%)	120	51 (42%)	62	26 (42%)	356	118 (33%)
**Non-participants**											
Age (years)		1130	28·3 (17·3–43·3)	883	29·3 (16·3–45·3)	596	30·3 (19·3–47·3)	355	25·3 (16·3–41·3)	2964	28·3 (17·3–44·3)
Weight (kg)		582	45·5 (28·0–53·0)	566	45·0 (25·0–53·0)	466	43·0 (30·0–49·0)	275	38·0 (20·0–47·0)	1889	43·0 (25·0–51·0)
Height (cm)		565	152 (132–160)	545	150 (126–158)	466	146 (133–152)	275	143 (120–150)	1851	148 (127–155)
Sex											
	Male	1144	568 (50%)	907	449 (50%)	600	281 (47%)	355	175 (49%)	3006	1473 (49%)
	Female	1144	576 (50%)	907	458 (50%)	600	319 (53%)	355	180 (51%)	3006	1533 (51%)

Of 356 cohort members, 218 (61%) remained afebrile, 94 (26%) had one episode of fever, 31 (9%) had two episodes, eight (2%) had three episodes, and five (1%) had four fever episodes during the 24-month study period. Parasite densities of febrile participants were higher than non-febrile participants (regression slope 1·4, 95% CI 0·23–2·51; appendix). The temperature difference was significant for mixed infections (p=0·006), but not for single species infections (appendix). 190 (53%) of 356 cohort members were treated with antimalarial drugs (DHA and piperaquine) during the study period. Cohort participants treated for *P falciparum* monoinfections, but not for the other species, had significantly higher parasite densities at the time of treatment (mean 652 497 per mL) than untreated cohort members (177 520 per ml; p=0·02). Cohort participants with an increasing number of episodes of parasitaemia were more likely to receive treatment (test for trend p<0·0001). Participants who tested positive only once for *P falciparum* had a 33% chance of being treated, whereas participants with 12 positive episodes had an 88% chance of being treated once or more. 178 (50%) of 356 individuals participated in all three rounds of the mass drug administration, 82 (24%) in two rounds, 50 (14%) in one round, and two (1%) did not participate in the mass drug administration.

Participants in the *P falciparum* group had between one and 13 uPCR tests positive for *P falciparum,* mixed *P falciparum* and *P vivax* (*P falciparum, P falciparum* and *P vivax* mixed), or unidentified species during the 24-month study period (appendix). The number of participants with multiple positive uPCR tests followed a logarithmic distribution; 76 (21%) of 354 participants had a single positive test, but only one had 13 positive tests (appendix). Participants of the *P vivax* groups had between one and 18 uPCR positive tests (appendix). The longer the infections lasted, the more likely the participants were to have a higher density infection. The geometric mean parasite density in participants in the P falciparum group with a single positive uPCR test was 1957 parasites per mL (95% CI 948–4041), whereas those with five positive uPCR tests had a geometric mean maximum parasite density of 96 679 parasites per mL (7500–1 246 310; test for trend p<0·0001).

The durations of 638 episodes of *Plasmodium* infections were assessed in 356 participants. The episodes lasted between 0 and 20 months (appendix).

**Figure 2:**
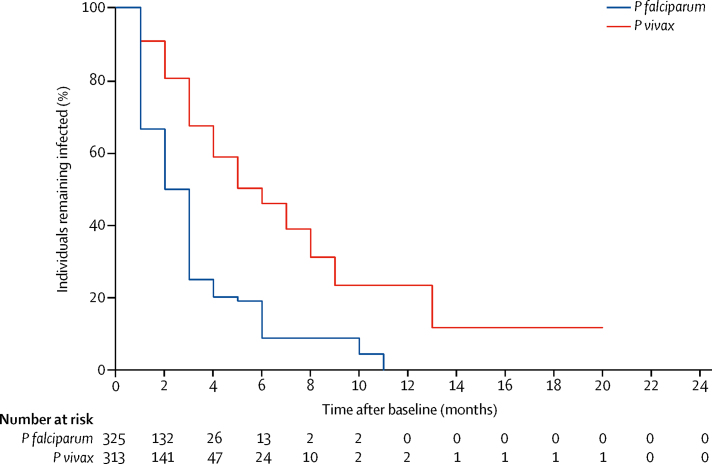
Persistence of *Plasmodium falciparum* and *Plasmodium vivax* infections over the study period *P falciparum* group infections included *P falciparum, P falciparum* and *P vivax* mixed infections, or unidentified spp. *P vivax* group infections included *P vivax, P vivax and P falciparum* mixed infections, or unidentified spp.

**Figure 3:**
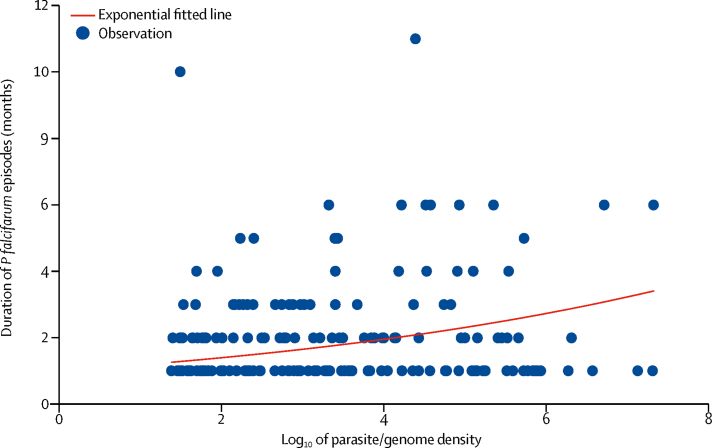
Correlation between the duration of a *Plasmodium falciparum* episode and parasite density Blue dots represent individuals; red line represents the trend line.

The median duration of *P falciparum* episodes was 2 months (IQR 1–3); and after accounting for censoring, participants had a 20% chance of having parasitaemia for 4 months or longer. There was no correlation between the age of participants and the duration of episodes (ρ=–0·005; p=0·9). The longest episode in the *P falciparum* group lasted for 11 months and the duration of the 10% longest persistent episodes ranged between 3 and 11 months. The median duration of *P vivax* was 6 months (IQR 3–9), and participants had a 59% chance of carrying detectable parasites for 4 months or longer. The longest *P vivax* group episode lasted for 20 months; the duration of the 10% most persistent episodes ranged from 5 to 20 months (figure 2). After accounting for censoring, individuals infected with *P falciparum* had a 9% chance of having parasitaemia for 4 months or longer. Individuals with *P vivax* infections had a 46% chance of having detectable parasites for 6 months or longer. A significant exponential relationship was observed between the parasite density and duration (figure 3). Higher densities of *P falciparum* and *P vivax* were associated with prolonged duration of infection (p<0·0001). The proportion of participants with long durations of plasmodium episodes (>5 months) was not significantly different in male and female participants (p=0·09). The age of participants with episodes of 6 months or longer were not significantly different in age from participants with episodes of shorter duration (for *P falciparum* p=0·5; for *P vivax* p=0·1).

**Figure 4:**
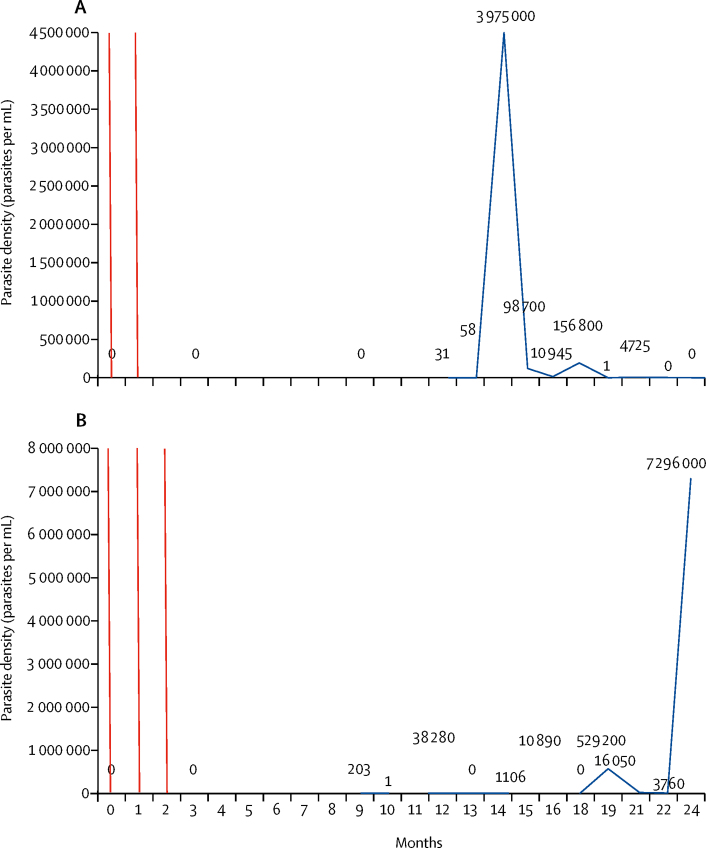
Examples of parasite densities over time in two individual cohort members (A) Participant VN300445: 12 months after two rounds of dihydroartemisinin and piperaquine a low-density *Plasmodium falciparum* infection (31 parasites per mL) was detected, which increased to 58 parasites per mL before expanding to 3 975 000 parasites per mL. The participant remained afebrile throughout the 2-year follow-up period and was not treated after the mass drug administration. (B) Participant VN300699: 9 months after three rounds of dihydroartemisinin and piperaquine, the participant had a low-density *P falciparum* infection. The density oscillates in the following 13 months between no detectable parasites and 529 200 parasites per mL to increase to 7 296 000 parasites per mL on the last study visit. The participant remained afebrile throughout the 2-year follow-up period and was not treated after the mass drug administration. Red lines represent mass drug administrations. Numbers on the graph represent parasite densities per mL. More individual examples are in the appendix.

**Figure 5:**
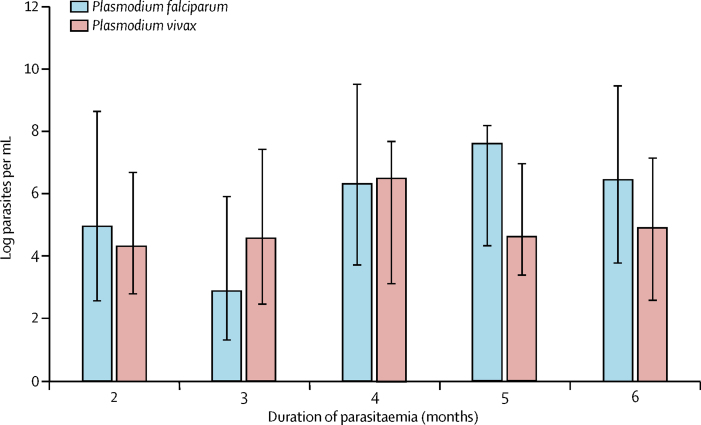
Amplitude of parasite density oscillations in relation to the observed duration of plasmodium infections Bars and whiskers are median and IQR.

Parasite densities oscillated over time, and ultralow-density infections frequently evolved into higher density infections. 16 (7%) of 222 untreated infections changed to a density of more than 200 000 parasites per mL. Variations in parasitaemia over time in two individuals with persisting *P falciparum* or *P vivax* infections are shown in figures 4A and 4B and additional cases are shown in the appendix. The amplitude in *P falciparum* densities varied with the duration of infection (figure 5). The median amplitude in *P falciparum* densities in individuals who had parasitaemia for 2 months was log 5·0 (IQR 2·5–8·7) and increased to log 6·5 (3·8–9·5) in individuals who had *P falciparum* for 6 months. *P vivax* densities increased from log 4·3 to log 6·5 in individuals who had parasitaemia for 2 months or 3 months or longer, but decreased to log 4·6 (4·3–8·2) in individuals who had *P vivax* for 5 months.

## Discussion

In this study, we found frequent persistent *P falciparum* and *P vivax* infections in a cohort of villagers in Vietnam. The median duration of *P falciparum* and *P vivax* infections was 1 month, but 9% of participants with *P falciparum* infections and 46% of participants with *P vivax* infections had infections lasting 6 months or longer. Overdispersion is a common feature of infectious disease transmission with a small proportion of the population contributing a large proportion of transmission. In the study area in Vietnam, the malaria transmission season is from September to March of the following year. Transmission is almost absent during the intervening months, so persistent carriage of parasites for 6 months or longer in a proportion of individuals could bridge this period and sustain malaria endemicity. The parasite densities of persistent infections varied widely (over six orders of magnitude); high-density infections developed after ultralow-density infections, which are undetectable by traditional diagnostic methods (including RDTs, microscopy, and standard PCR). This pattern was observed for both *P falciparum* and *P vivax* infections, with parasite densities varying between 10^2^ and 10^8^ times over time. A significant correlation between parasite density and duration of infection was observed for *P falciparum* and *P vivax*. The probability that a high-density infection was detected during persistent infections increased with the number of observations. 7% of the untreated ultralow infections escalated into high-density infections that threaten the host, as well as the community because of their transmissibility. An untransmissible infection at one time could become a transmissible infection weeks later. It is difficult to imagine that malaria parasites have evolved sophisticated mechanisms to ensure long-term persistence in their hosts if this did not translate into a transmission advantage.

The extended persistence of *P vivax* infections is well known. *P vivax* hypnozoites trigger relapsing infections that can be cleared by radical cure, which must include a full course of an 8-aminoquinoline (eg, primaquine). Although the persistence of *P falciparum* infections is less well described, especially in low-transmission settings, there is an extensive literature from malaria therapy of neurosyphilis suggesting that persistent infections for longer than a year are not unusual.^14^, ^15^, ^16^, ^17^ Persistent *P falciparum* infections of up to 13 years have been reported.^18^ To accelerate malaria elimination, targeting the asymptomatic parasite reservoir is likely to be of crucial importance.

Previous studies of persistent *P falciparum* and *P vivax* infections, most notably studies of malaria therapy,^14^, ^15^, ^16^, ^19^, ^20^, ^21^, ^22^, ^23^, ^24^ differ in several key aspects from our study in Vietnamese villagers. Past studies used light microscopy, which detect parasite densities above 50 000 parasites per mL, or standard rtPCR, which detects parasite densities above 1000 parasites per mL. The lower limit of the detection of uPCR used in the current study was 22 per mL. Second, the timing of the initial infection was well defined in malaria therapy studies, but is unknown in this study. Third, it seems reasonable to assume that the initial inoculation in malaria therapy was a single clone, whereas naturally acquired infections in this study are often polyclonal and might require longer to control through an immune response. Fourth, more than half of the cohort population were treated with curative antimalarial therapy whenever an infection was detected frequently interrupting persistent infections. By contrast with the therapeutic malaria trials, patients only received treatment when more severe disease developed and then only with the aim to reduce parasitaemia rather than cure. Finally, few, if any, of the patients treated with malaria for neurosyphilis and other disorders in the USA had previous natural exposure to *Plasmodium* spp infections, and therefore did not have immunity. The initial inoculation of *P falciparum* or *P vivax* in malaria therapy tended to result in high parasite densities, which patients controlled with or without antimalarial treatment (eg, quinine). Conversely, the Vietnamese study population has been previously exposed to plasmodium infections potentially resulting in premunition. In this pre-exposed population, ultralow-density infections can persist undetected for extended periods followed by wide oscillations above 10 million parasites per mL.

This study has several limitations. First, cohort members were sampled at monthly intervals. Parasite density changes that occur more frequently could have been missed. Ultralow-density infections can take 2–3 weeks to reach high densities detectable by standard diagnostic tools. Thus, most large oscillations in parasite densities can probably be detected by monthly surveys. Second, the preceding period of carriage could not be assessed in individuals with parasitaemia identified at the first survey, which could have caused underestimation of the period of carriage reported in this study. Third, based on the available data, it is not possible to exclude serial reinfections as a cause of the observed persistence in parasitaemia. However, this is unlikely in a very low endemicity setting such as the study sites. Genotyping was not possible due to insufficient quantities of parasite nucleic acid. Fourth, treatment of symptomatic participants required censoring as artemisinin-based combination therapies permanently clears susceptible schizontocytes. Treatment-seeking behaviour, prescription practices, and adherence to follow-up vary between communities and regions limiting the generalisability of the study findings.

20 years ago, Farnert and colleagues found daily changes in parasite densities determined by microscopy over a 14-day follow-up period in rural Tanzania.^25^ Findings from a study^26^ in Pailin, western Cambodia, showed much shorter persistence of *P falciparum* infections than this study. However, in the Pailin study, the number of *P falciparum*-infected individuals was low (n=32), and initial parasitaemias were lower (median parasite density baseline Pailin 335 per mL, Vietnam 465 per mL). Undocumented treatment can also not be excluded in the Pailin study site.^27^ Additionally, the genetic structure of the local parasite populations might have a role.^28^ Persistence of *Plasmodium* spp infections depends on the evasion of adaptive immunity by antigenic variation over time.^29^ Also, the very low transmission of *P falciparum* in Pailin province, with only a few parasite strains circulating, might increase the likelihood of strain-specific immunity in the host, facilitating parasite clearance without antimalarial treatment.

In conclusion, a pattern of persistent *P falciparum* and *P vivax* infections with oscillating densities was observed in this study population over 2 years of follow-up. The submicroscopic, asymptomatic plasmodium reservoir might have a crucial role in sustaining transmission of malaria and probably explains the seasonal malaria increase after the dry season in settings like southern Vietnam. Screening interventions that rely on standard diagnostic tools such as RDTs will not identify most of the asymptomatic low-density plasmodium reservoir. Even highly sensitive tests will not identify all parasitaemic individuals. Targeting the asymptomatic plasmodium reservoir is likely to be important to accelerate malaria elimination.

**This online publication has been corrected. The corrected version first appeared at thelancet.com/infection on April 19, 2018**
