# Corrections

**DOI:** 10.1016/S1473-3099(18)30236-6

**Published:** 2018-05

*Nguyen T-N, von Seidlein L, Nguyen T-V, et al. The persistence and oscillations of submicroscopic Plasmodium falciparum and Plasmodium vivax infections over time in Vietnam: an open cohort study.* Lancet Infect Dis *2018; **18:** 565–72—*In this Article, the lowest parasite densities shown in figure 3 were found to be below the lower limit of accurate quantification of the PCR method used in the study (22 parasites per mL). The analysis was therefore redone after excluding these low parasite densities. The overall findings remain unchanged. The end of the third paragraph in the Results section has been amended to state: The geometric mean parasite density in participants in the *P falciparum* group with a single positive uPCR test was 1957 parasites per mL (95% CI 948–4041), whereas those with five positive uPCR tests had a geometric mean maximum parasite density of 96 679 parasites per mL (7500–1 246 310; test for trend p<0·0001). Figure 3 has also been updated. These corrections have been made to the online version as of April 19, 2018, and the printed Article is correct.

