# Supplementary material for: The persistence and oscillations of submicroscopic Plasmodium falciparum and Plasmodium vivax infections over time in Vietnam: an open cohort study
Source: Lancet Infect Dis. 2018 May;18(5):565–72. doi: 10.1016/S1473-3099(18)30046-X (PMC5910058; doi:10.1016/S1473-3099(18)30046-X)
Supplement: Supplementary appendix [file mmc1.pdf]

THE LANCET Infectious Diseases

# Supplementary appendix

This appendix formed part of the original submission and has been peer reviewed. We post it as supplied by the authors.

Supplement to: Nguyen T-N, von Seidlein L, Nguyen T-V, et al. The persistence and oscillations of submicroscopic Plasmodium falciparum and *Plasmodium vivax* infections over time in Vietnam: an open cohort study. *Lancet Infect Dis* 2018; published online Jan 22. http://dx.doi.org/10.1016/S1473-3099(18)30046-X.

# The persistence and oscillations of submicroscopic *Plasmodium falciparum* and *vivax* infections over time in Vietnam: an open cohort study - Appendix

## Tables

Table S1: The frequency of *P. falciparum*, *Pf/Pv* mixed or *Pspp*. infections in relation to PCR test performed

Table S2: The frequency of *P. vivax, Pf/Pv* mixed or *Pspp*. Infections in relation to PCR test performed

Table S3: The duration of 638 episodes of Plasmodium infections

## Figures

Figure S1: Venn diagram illustrating *P falciparum* and *P vivax* infection groups

Figure S2: The relationship of parasite density (log scale) and fever for all parasitaemias

Figure S3: The distribution of the number of surveys that the study cohort members participated in.

Figure S4: Number of group members who had uPCR tests positive for *Pf, Pf/Pv mixed,* or *P.spp* (with trendline)

Figure S5: The maximum parasite density (log scale) increases with duration of *Pf, Pf/Pv mixed,* or *P.spp* (with linear trendline).

Figure S6: *Pf, Pf/Pv* or *P. spp* infections - MDA on M0, M1 and M2

Figure S7: *Pf, Pf/Pv* or *P. spp* infections - MDA on M12, M13 and M14

Figure S8: *Pv, Pf/Pv* or *P. spp* infections - MDA on M0, M1 and M2

Figure S9: *Pv, Pf/Pv* or *P. spp* infections - MDA on M12, M13 and M14

**Table S1: The frequency of *P. falciparum*, *Pf/Pv* mixed or *Pspp*. infections in relation to PCR test performed**

| Number of PCR tests performed/ cohort member | Total tests performed | People tested | Number of infections | | | | | | | | | | | | | | Total positives | Positive tests /member | % tests positive |
|---|---|---|---|---|---|---|---|---|---|---|---|---|---|---|---|---|---|---|---|
| | | | 0 | 1 | 2 | 3 | 4 | 5 | 6 | 7 | 8 | 9 | 10 | 11 | 12 | 13 | | | |
| 0 | 0 | 0 | 0 | | | | | | | | | | | | | | 0 | | |
| 1 | 1 | 1 | 0 | 1 | | | | | | | | | | | | | 1 | 1.0 | 100% |
| 2 | 14 | 7 | 0 | 6 | 1 | | | | | | | | | | | | 8 | 1.1 | 57% |
| 3 | 6 | 2 | 0 | 0 | 2 | | | | | | | | | | | | 4 | 2.0 | 67% |
| 4 | 32 | 8 | 0 | 6 | 1 | 1 | | | | | | | | | | | 11 | 1.4 | 34% |
| 5 | 40 | 8 | 0 | 4 | 2 | 1 | 1 | | | | | | | | | | 15 | 1.9 | 38% |
| 6 | 96 | 16 | 0 | 6 | 4 | 3 | 2 | 1 | | | | | | | | | 36 | 2.3 | 38% |
| 7 | 84 | 12 | 0 | 4 | 3 | 2 | 1 | 2 | | | | | | | | | 30 | 2.5 | 36% |
| 8 | 200 | 25 | 0 | 5 | 5 | 5 | 6 | 3 | 0 | 1 | | | | | | | 76 | 3.0 | 38% |
| 9 | 180 | 20 | 0 | 5 | 3 | 4 | 3 | 3 | 2 | | | | | | | | 62 | 3.1 | 34% |
| 10 | 270 | 27 | 0 | 6 | 5 | 6 | 1 | 5 | 1 | 0 | 2 | 1 | | | | | 94 | 3.5 | 35% |
| 11 | 264 | 24 | 0 | 6 | 6 | 3 | 3 | 1 | 2 | 2 | 1 | | | | | | 78 | 3.3 | 30% |
| 12 | 612 | 51 | 0 | 9 | 13 | 8 | 4 | 5 | 5 | 1 | 3 | 2 | 1 | | | | 189 | 3.7 | 31% |
| 13 | 390 | 30 | 0 | 4 | 6 | 3 | 3 | 5 | 2 | 2 | 2 | 2 | 0 | 0 | 1 | | 134 | 4.5 | 34% |
| 14 | 406 | 29 | 0 | 3 | 8 | 5 | 1 | 2 | 0 | 4 | 2 | 3 | 0 | 0 | 0 | 1 | 132 | 4.6 | 33% |
| 15 | 300 | 20 | 0 | 3 | 3 | 1 | 3 | 1 | 1 | 4 | 1 | 0 | 1 | 0 | 2 | | 105 | 5.3 | 35% |
| 16 | 304 | 19 | 0 | 2 | 1 | 3 | 3 | 2 | 0 | 3 | 1 | 0 | 2 | 1 | 1 | | 107 | 5.6 | 35% |
| 17 | 221 | 13 | 0 | 4 | 1 | 1 | 2 | 0 | 2 | 2 | 0 | 1 | | | | | 52 | 4.0 | 24% |
| 18 | 198 | 11 | 0 | 0 | 1 | 1 | 2 | 2 | 1 | 1 | 0 | 3 | | | | | 63 | 5.7 | 32% |
| 19 | 190 | 10 | 0 | 1 | 1 | 2 | 1 | 0 | 1 | 2 | 0 | 0 | 0 | 1 | 1 | | 56 | 5.6 | 29% |
| 20 | 240 | 12 | 0 | 1 | 1 | 2 | 2 | 2 | 1 | 0 | 0 | 1 | 1 | 1 | | | 63 | 5.3 | 26% |
| 21 | 147 | 7 | 0 | 0 | 1 | 0 | 1 | 0 | 1 | 3 | 1 | | | | | | 41 | 5.9 | 28% |
| 22 | 44 | 2 | 0 | 0 | 0 | 0 | 1 | 0 | 1 | | | | | | | | 10 | 5.0 | 23% |
| Total | 4239 | 354 | 0 | 76 | 68 | 51 | 40 | 34 | 20 | 25 | 13 | 13 | 5 | 3 | 5 | 1 | 1367 | 3.9 | 32% |

**Table S2: The frequency of *P. vivax, Pf/Pv* mixed or *Pspp*. Infections in relation to PCR test performed**

| Number of PCR tests performed/ cohort member | Total tests performed | People tested | Number of infections | | | | | | | | | | | | | | | | | | | Total positives | Positive tests /member | % tests positive |
|---|---|---|---|---|---|---|---|---|---|---|---|---|---|---|---|---|---|---|---|---|---|---|---|---|
| | | | 0 | 1 | 2 | 3 | 4 | 5 | 6 | 7 | 8 | 9 | 10 | 11 | 12 | 13 | 14 | 15 | 16 | 17 | 18 | | | |
| 0 | 0 | 0 | 0 | | | | | | | | | | | | | | | | | | | 0 | | |
| 1 | 1 | 1 | 0 | 1 | | | | | | | | | | | | | | | | | | 1 | 1.0 | 100% |
| 2 | 4 | 2 | 0 | 2 | | | | | | | | | | | | | | | | | | 2 | 1.0 | 50% |
| 3 | 3 | 1 | 0 | 0 | 1 | | | | | | | | | | | | | | | | | 2 | 2.0 | 67% |
| 4 | 20 | 5 | 0 | 1 | 2 | 1 | 1 | | | | | | | | | | | | | | | 12 | 2.4 | 60% |
| 5 | 20 | 4 | 0 | 2 | 0 | 1 | 1 | | | | | | | | | | | | | | | 9 | 2.3 | 45% |
| 6 | 72 | 12 | 0 | 3 | 2 | 2 | 2 | 3 | | | | | | | | | | | | | | 36 | 3.0 | 50% |
| 7 | 56 | 8 | 0 | 0 | 1 | 1 | 3 | 2 | 1 | | | | | | | | | | | | | 33 | 4.1 | 59% |
| 8 | 152 | 19 | 0 | 6 | 4 | 5 | 1 | 1 | 2 | | | | | | | | | | | | | 50 | 2.6 | 33% |
| 9 | 144 | 16 | 0 | 5 | 0 | 1 | 3 | 5 | 1 | 1 | | | | | | | | | | | | 58 | 3.6 | 40% |
| 10 | 210 | 21 | 0 | 5 | 4 | 0 | 2 | 3 | 2 | 1 | 4 | | | | | | | | | | | 87 | 4.1 | 41% |
| 11 | 165 | 15 | 0 | 2 | 1 | 2 | 1 | 3 | 1 | 2 | 1 | 1 | 1 | | | | | | | | | 76 | 5.1 | 46% |
| 12 | 492 | 41 | 0 | 17 | 4 | 2 | 5 | 3 | 1 | 3 | 1 | 2 | 2 | 1 | | | | | | | | 150 | 3.7 | 30% |
| 13 | 299 | 23 | 0 | 6 | 0 | 1 | 1 | 0 | 0 | 3 | 5 | 1 | 3 | 1 | 1 | 1 | | | | | | 149 | 6.5 | 50% |
| 14 | 280 | 20 | 0 | 5 | 5 | 2 | 1 | 0 | 0 | 1 | 2 | 1 | 2 | 1 | | | | | | | | 88 | 4.4 | 31% |
| 15 | 255 | 17 | 0 | 4 | 2 | 2 | 1 | 0 | 1 | 2 | 0 | 2 | 2 | 0 | 1 | | | | | | | 88 | 5.2 | 35% |
| 16 | 256 | 16 | 0 | 3 | 1 | 1 | 3 | 1 | 2 | 1 | 1 | 1 | 0 | 1 | 0 | 1 | | | | | | 85 | 5.3 | 33% |
| 17 | 153 | 9 | 0 | 2 | 0 | 1 | 2 | 0 | 1 | 0 | 1 | 0 | 0 | 1 | 0 | 1 | | | | | | 51 | 5.7 | 33% |
| 18 | 162 | 9 | 0 | 1 | 0 | 0 | 0 | 3 | 1 | 1 | 0 | 1 | 0 | 0 | 1 | 0 | 1 | | | | | 64 | 7.1 | 40% |
| 19 | 190 | 10 | 0 | 2 | 3 | 0 | 1 | 0 | 0 | 0 | 1 | 0 | 1 | 0 | 0 | 0 | 1 | 0 | 0 | 0 | 1 | 63 | 6.3 | 33% |
| 20 | 220 | 11 | 0 | 2 | 2 | 2 | 1 | 3 | 0 | 1 | | | | | | | | | | | | 38 | 3.5 | 17% |
| 21 | 147 | 7 | 0 | 1 | 0 | 3 | 0 | 0 | 1 | 0 | 0 | 0 | 0 | 1 | 0 | 0 | 1 | | | | | 41 | 5.9 | 28% |
| 22 | 44 | 2 | 0 | 0 | 0 | 0 | 0 | 1 | 0 | 1 | | | | | | | | | | | | 12 | 6.0 | 27% |
| Total | 3345 | 269 | 0 | 70 | 32 | 27 | 29 | 28 | 14 | 17 | 16 | 9 | 11 | 6 | 3 | 3 | 3 | 0 | 0 | 0 | 1 | 1195 | 4.4 | 36% |

**Table S3: The duration of 638 episodes of Plasmodium infections**

| Group | n | Duration (IQR) in months | Range |
|---|---|---|---|
| ***P. falciparum**, Pf/Pv mixed or Pspp* | | | |
| 90% to 100% (longest duration) | 32 | 4(3-6) | (3-11) |
| 75% to 90% | 49 | 2(2-2) | (2-3) |
| 50% to 75% | 82 | 1(1-1) | (1-2) |
| 0% to 50% (shortest duration) | 162 | 0(0-1) | (0-1) |
| All (0% to 100%) | 325 | 1(0-2) | (0-11) |
| ***P. vivax**, Pf/Pv mixed or Pspp* | | | |
| 90% to 100% (longest duration) | 31 | 6(6-8) | (5-20) |
| 75% to 90% | 47 | 3(3-4) | (3-5) |
| 50% to 75% | 78 | 2(2-2) | (1-3) |
| 0% to 50% (shortest duration) | 157 | 1(1-1) | (0-1) |
| All (0% to 100%) | 313 | 1(1-3) | (0-20) |
| Pf +Pv (0% to 100%) | 638 | 1(1-2) | (0-20) |

**Figure S1:** Venn diagram illustrating *P. falciparum* and *P.vivax* infection groups

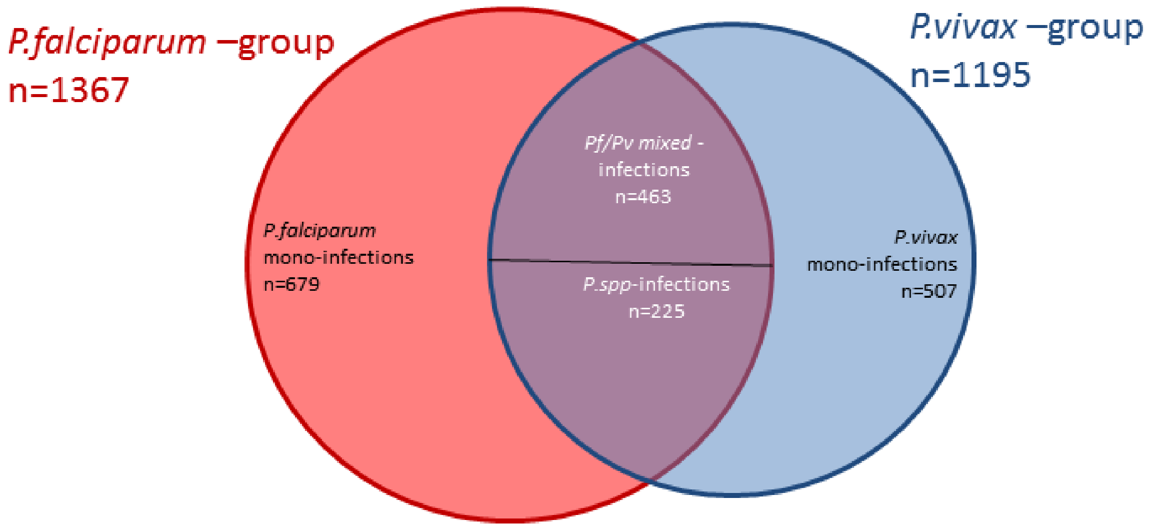

**Figure S2**: The relationship of parasite density (log scale) and fever for a) all parasitaemias b) *P falciparum* mono-infections and c) for *P vivax* mono-infections

a) all parasitaemias

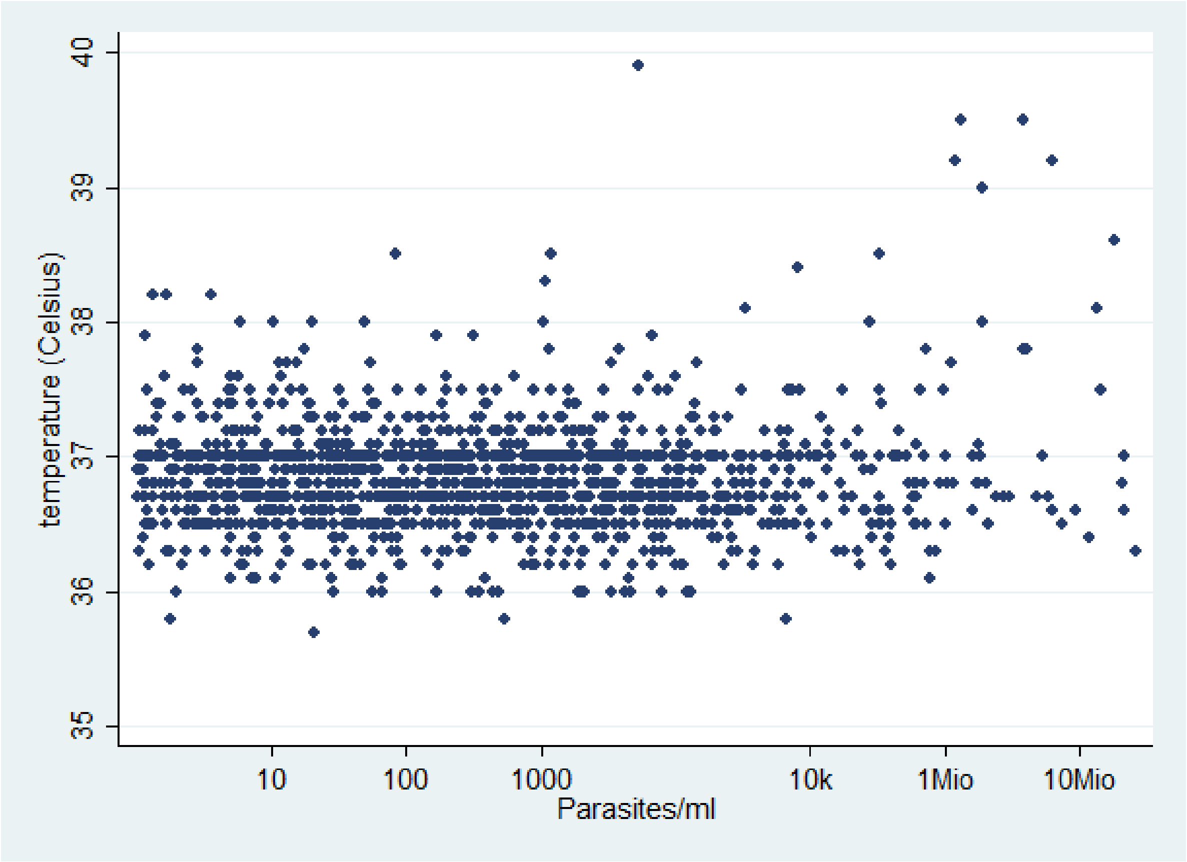


b) for *P falciparum* mono-infections

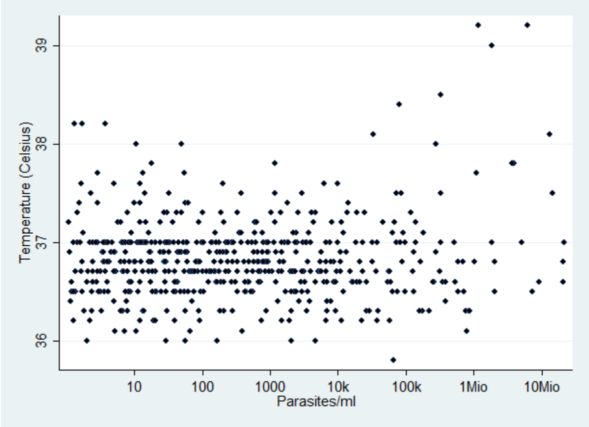


c) for *P vivax* mono-infections

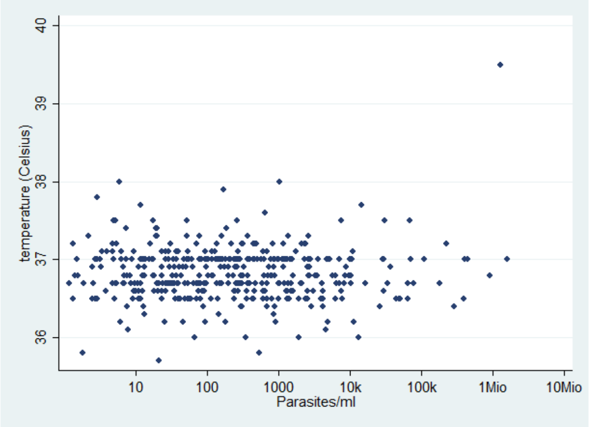

**Figure S3:** The distribution of the number of surveys that the study cohort members participated in.

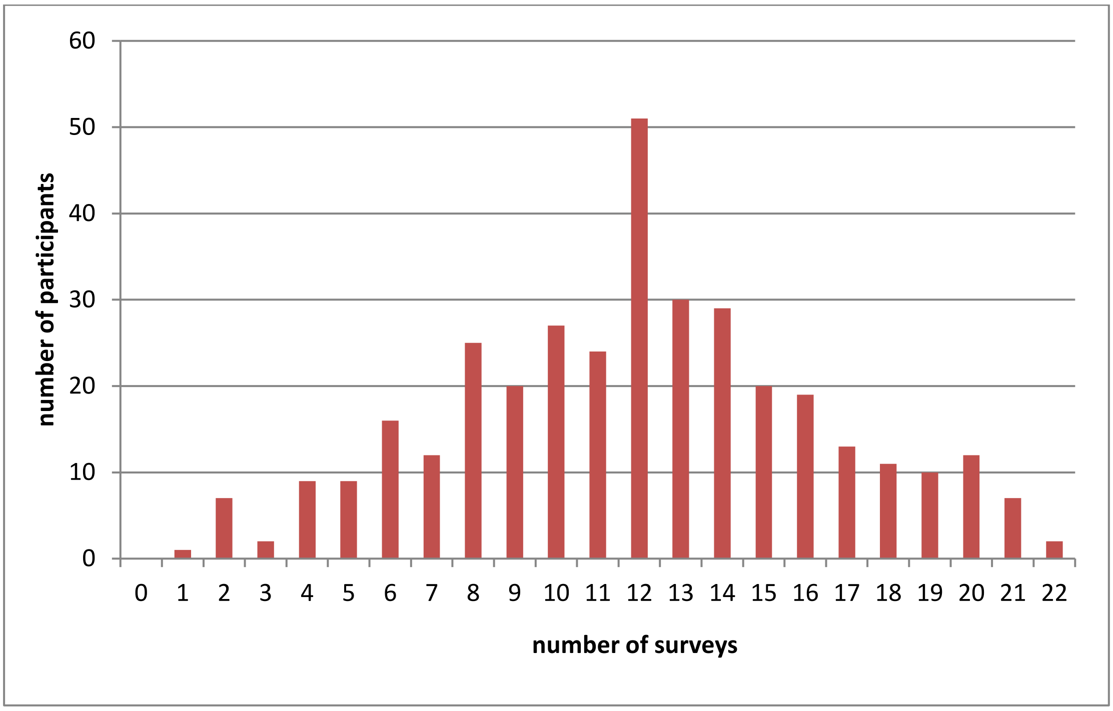

**Figure S4: Number of group members who had uPCR tests positive for *Pf, Pf/Pv mixed,* or *P.spp*** (with trendline)

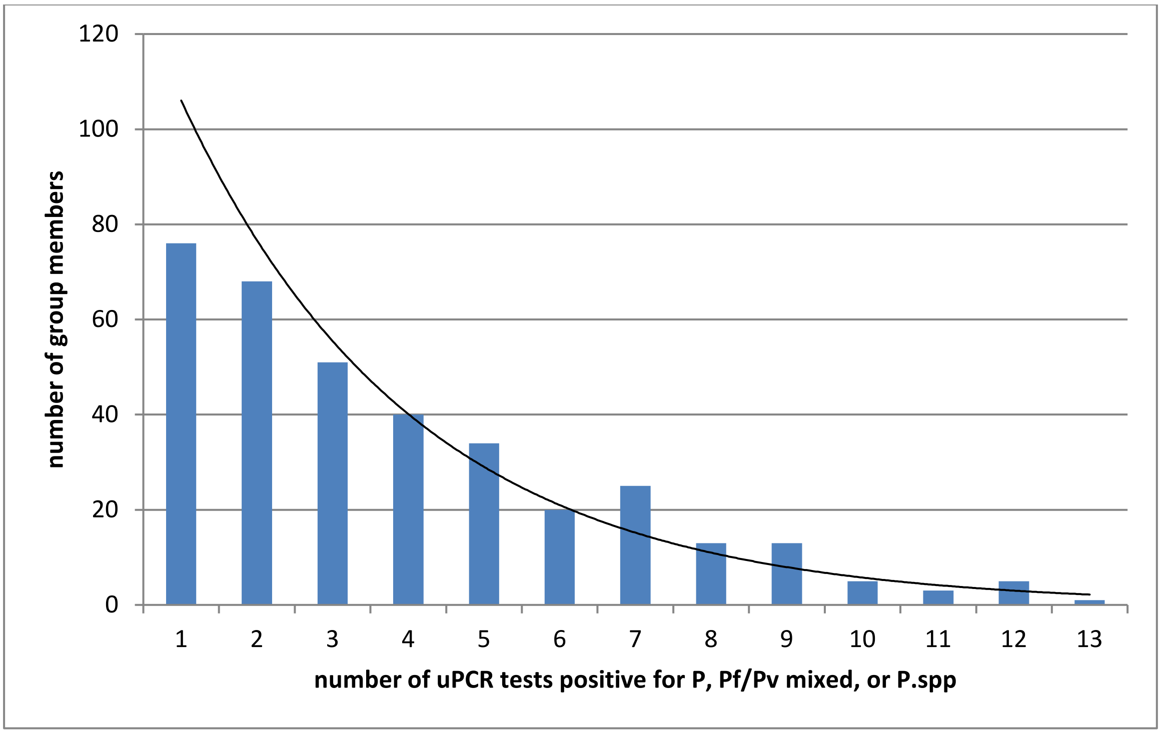

**Figure S5: The maximum parasite density (log scale) increases with duration of** ***Pf, Pf/Pv mixed,*** **or** ***P.spp*** (with linear trendline).

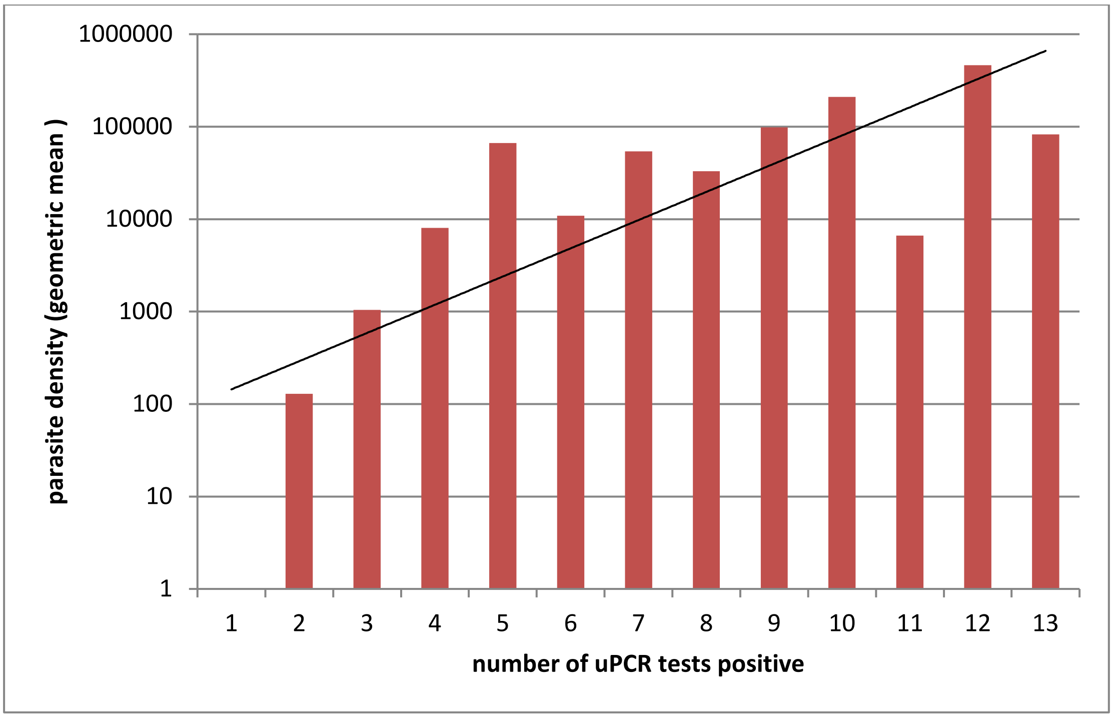

**Figure S6: *Pf, Pf/Pv* or *P. spp* infections - MDA on M0, M1 and M2**

A case series illustrating changes in parasite densities over time in 8 participants with *Pf, Pf/Pv* or *P. spp* infections residing in villages randomised to receive MDA on M0, M1 and M2. The number and timing of MDA(s) received by the participant is indicated by red lines. Parasite densities are provided above the respective month when the sample was collected. A febrile episode is indicated by a red star.

1 VN300493 GIA, Pf 13 episodes, MDA rounds 0, Fever (Fx) 1, Treatment (Rx) 0

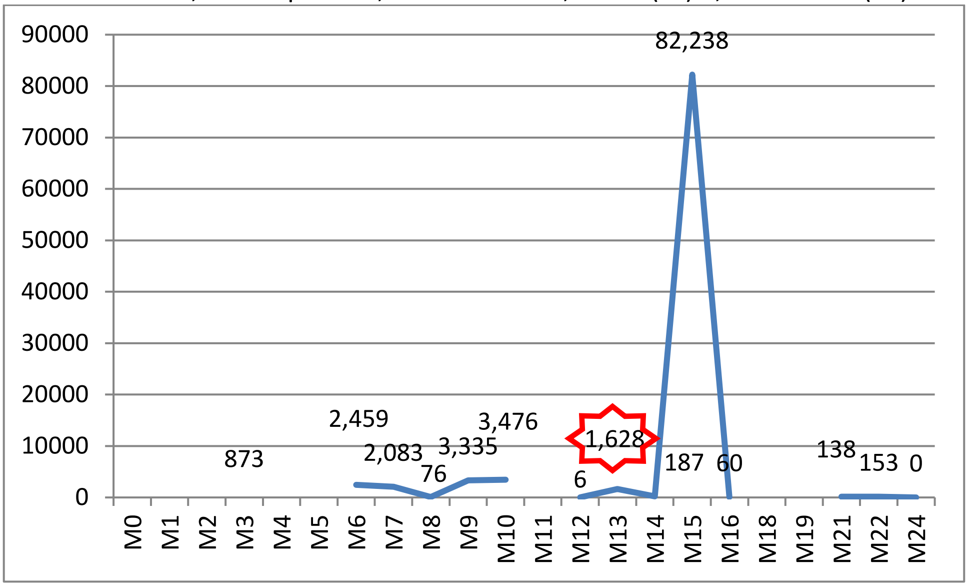


2 VN300694 GIA, MDA 2, Pf 12 episodes, Fx 0, Rx 0

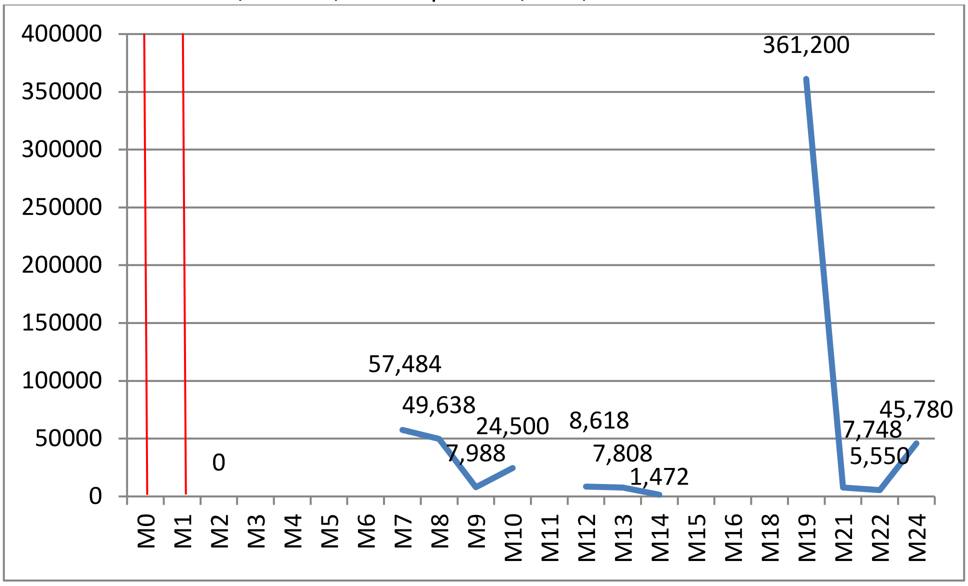

3 VN300192 GIA, MDA 3, Pf 11 episodes, Fx 1, Rx 0

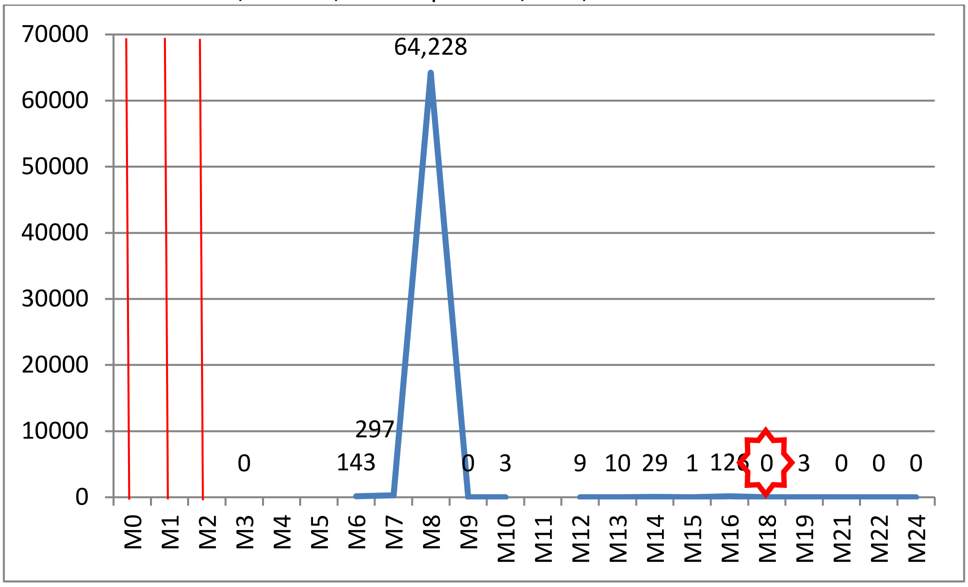


4 VN300405 GIA, MDA 3, Pf 10 episodes , Fx 2, Rx 0

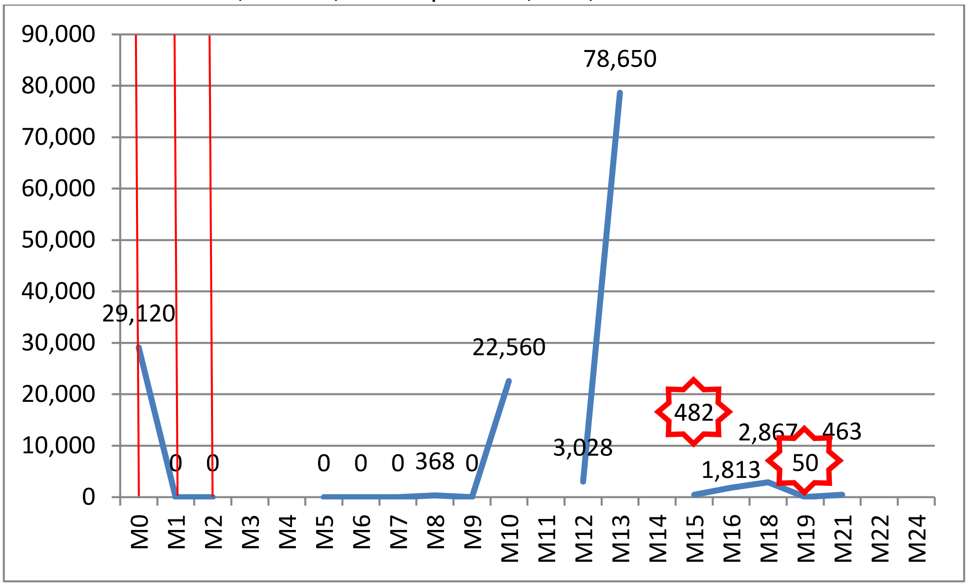


5 VN300699 GIA, MDA 3, Pf 9 episodes, Fx0, Rx 0

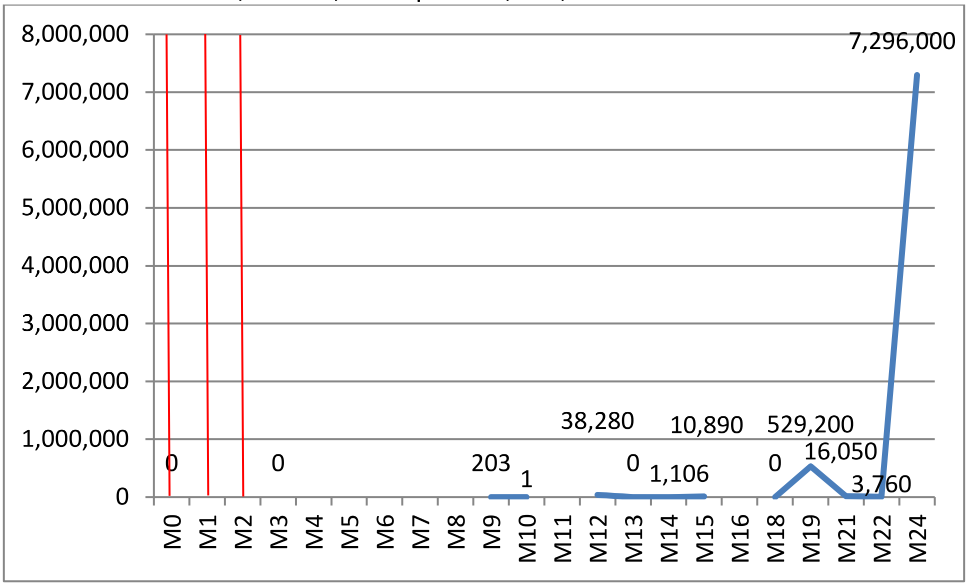

6 VN300445 GIA, MDA 2, Pf 9 episodes, Fx 3, Rx 0

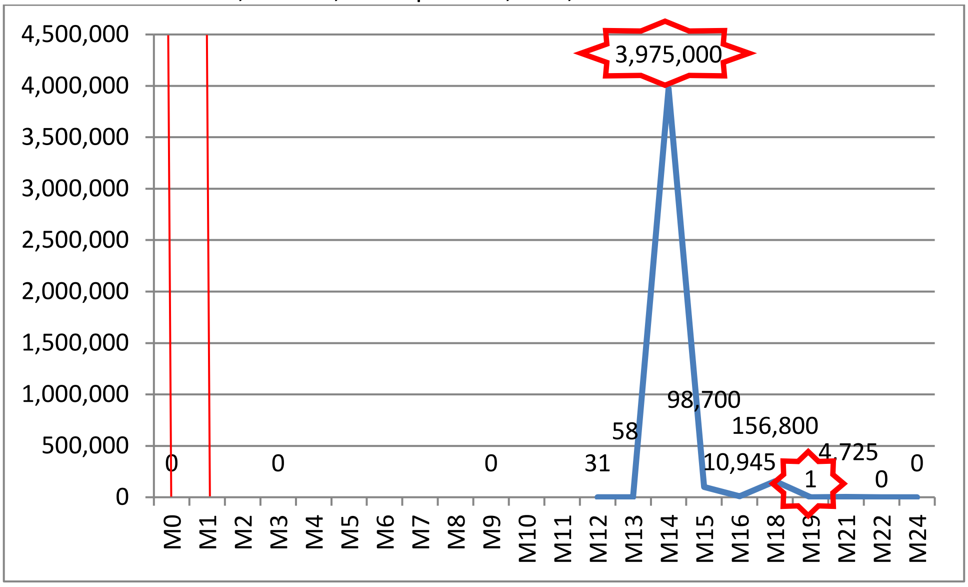


7 VN300443 GIA, MDA 3, Pf 9 episodes, Fx 0, Rx 0

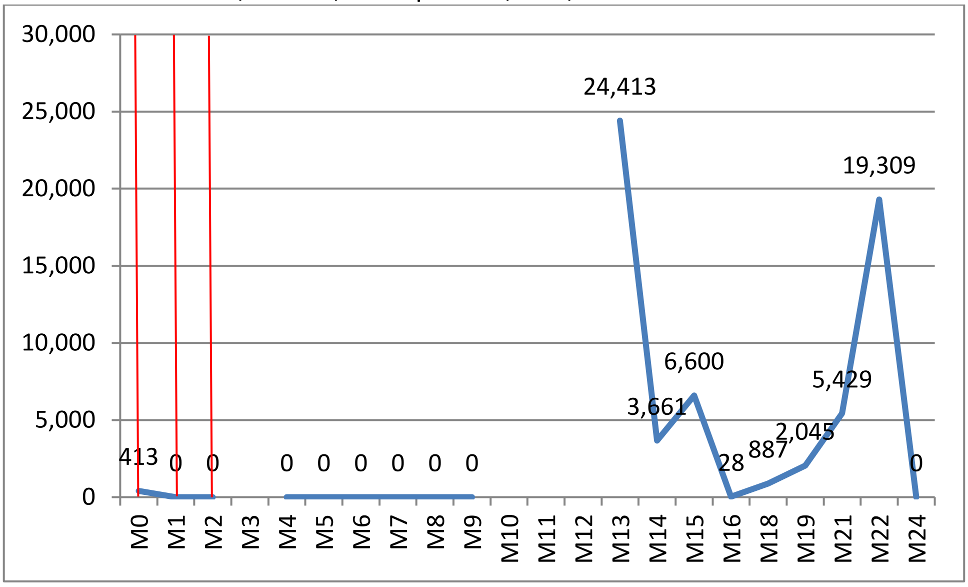


8 VN300485 GIA, MDA 3, Pf9 episodes, Fx 0, Rx 0

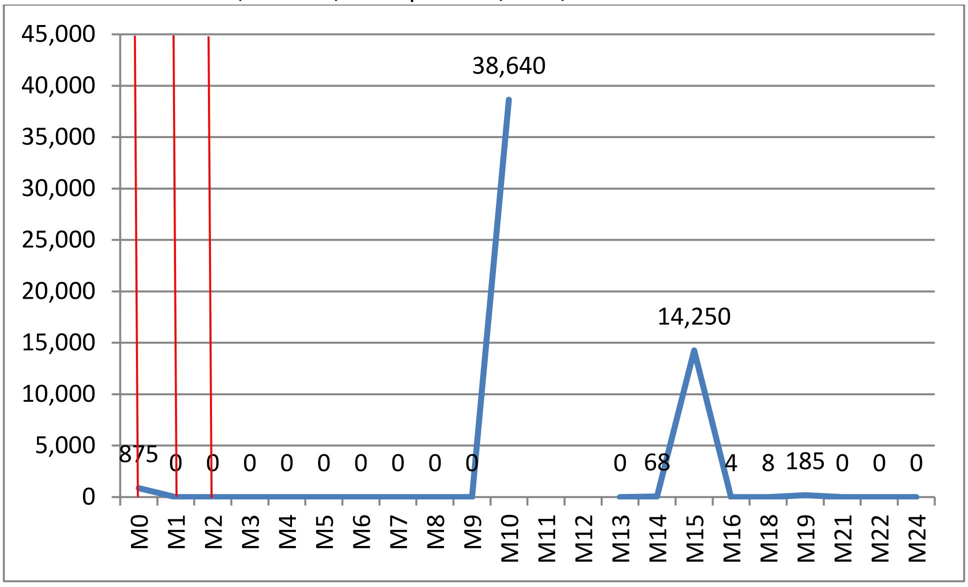

Legend:

GIA = Village
MDA = Mass drug administration
Pf = *Plasmodium falciparum*
Fx = Fever episodes
Rx = Antimalarial treatment received

**Figure S7: *Pf, Pf/Pv* or *P. spp* infections - MDA on M12, M13 and M14**

A case series illustrating changes in parasite densities over time in 4 participants with *Pf, Pf/Pv* or *P. spp* infections residing in villages randomised to receive MDA on M12, M13 and M14. The number and timing of MDA(s) received by the participant is indicated by red lines. Parasite densities are provided above the respective month when the sample was collected. A febrile episode is indicated by a red star.

1 VN400301 THA, MDA2, Pf 10 episodes, Fx 1, Rx 0

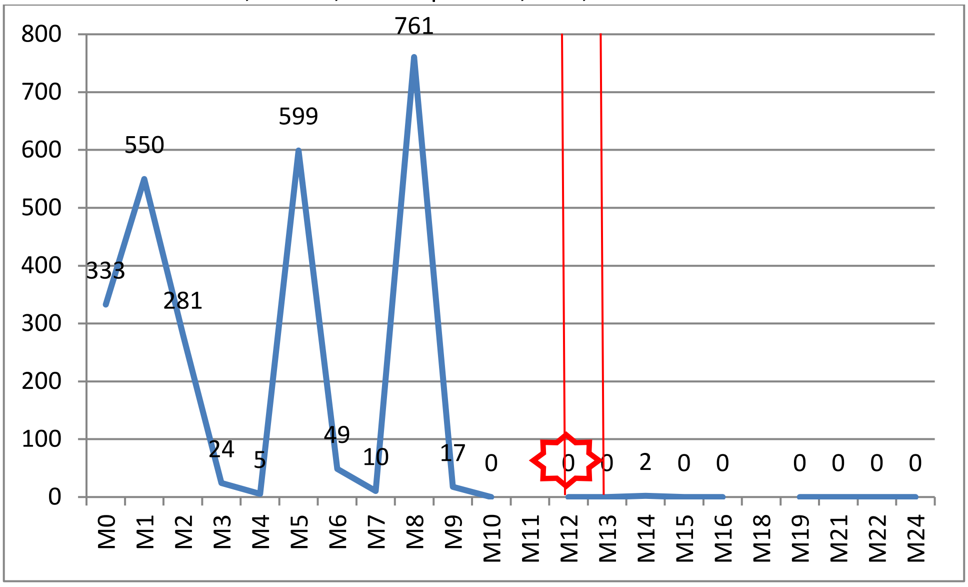


2 VN400093 THA, MDA 1, Pf 9 episodes, Fx 0, Rx 0

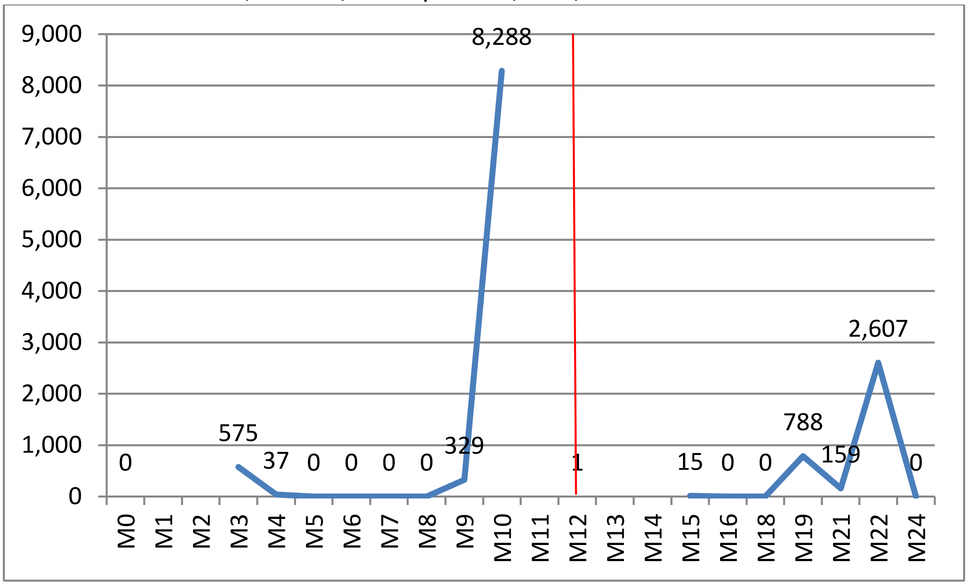

3 VN400083 THA, MDA 2, Pf 8 episodes, Fx 0, Rx 0

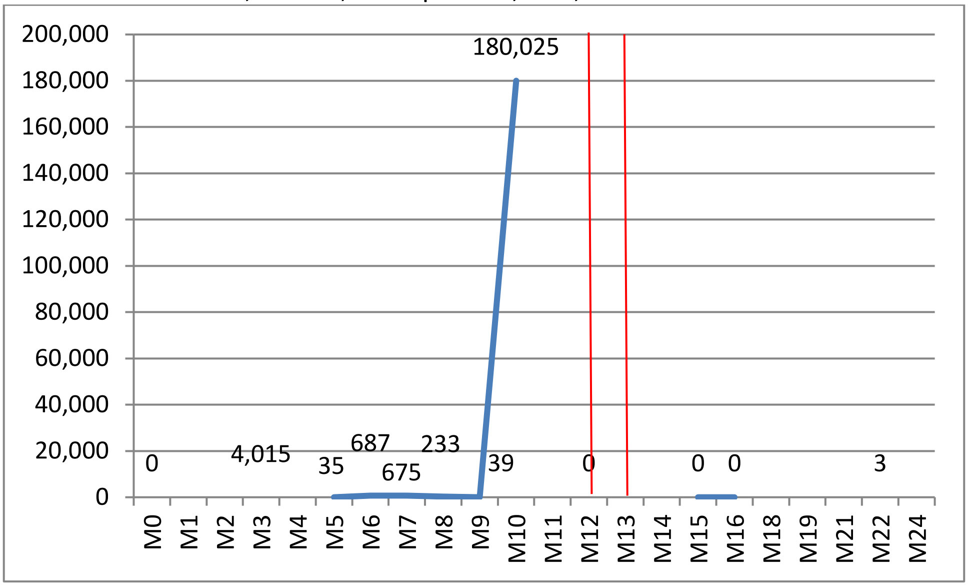


4 VN400380 THA, MDA 2, Pf 8 episodes, Fx 3, Rx 0

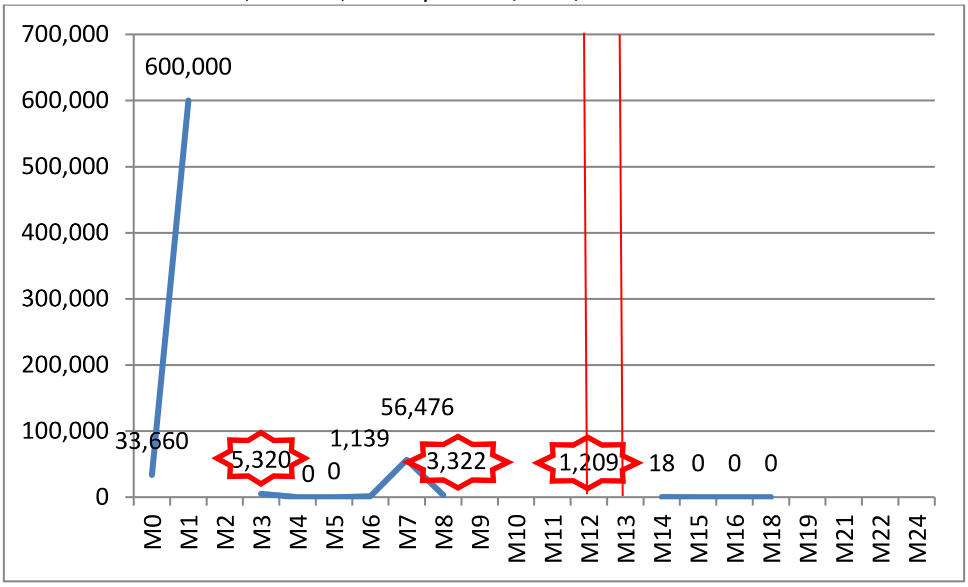


Legend:

THA = Village
MDA = Mass drug administration
Pf = *Plasmodium falciparum*
Fx = Fever episodes
Rx = Antimalarial treatment received

**Figure S8: *Pv, Pf/Pv* or *P. spp* infections - MDA on M0, M1 and M2**

A case series illustrating changes in parasite densities over time in 10 participants with *Pv, Pf/Pv* or *P. spp* infections residing in villages randomised to receive MDA on M0, M1 and M2. The number and timing of MDA(s) received by the participant is indicated by red lines. Parasite densities are provided above the respective month when the sample was collected. A febrile episode is indicated by a red star.

1 VN300711 GIA, MDA 3, Pv 14 episodes, Fx 2, Rx 0

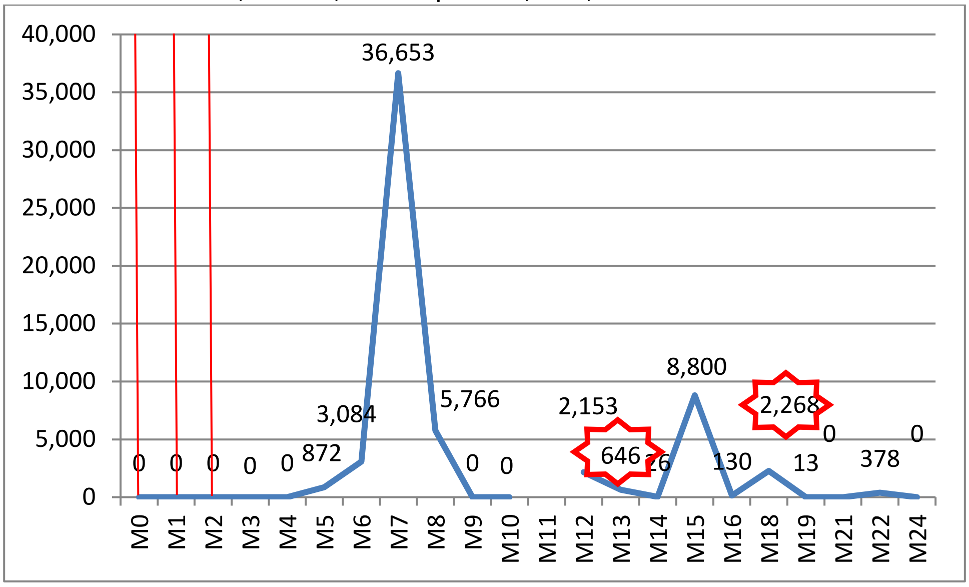


2 VN300694 GIA, MDA 2, Pv 13 episodes, Fx0, Rx 0

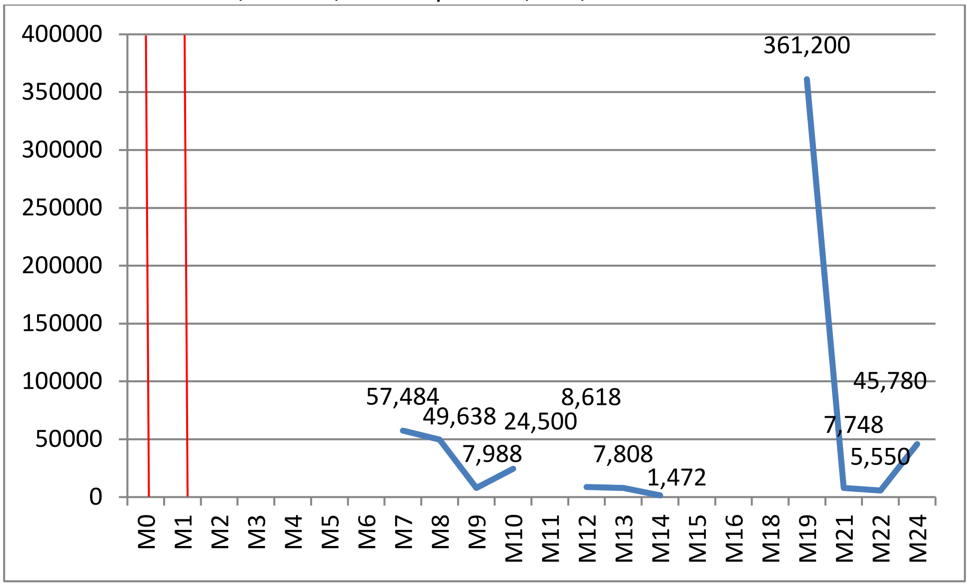

3 VN100356 BK, MDA 1, Pv 11 episodes, Fx 0, Rx 0

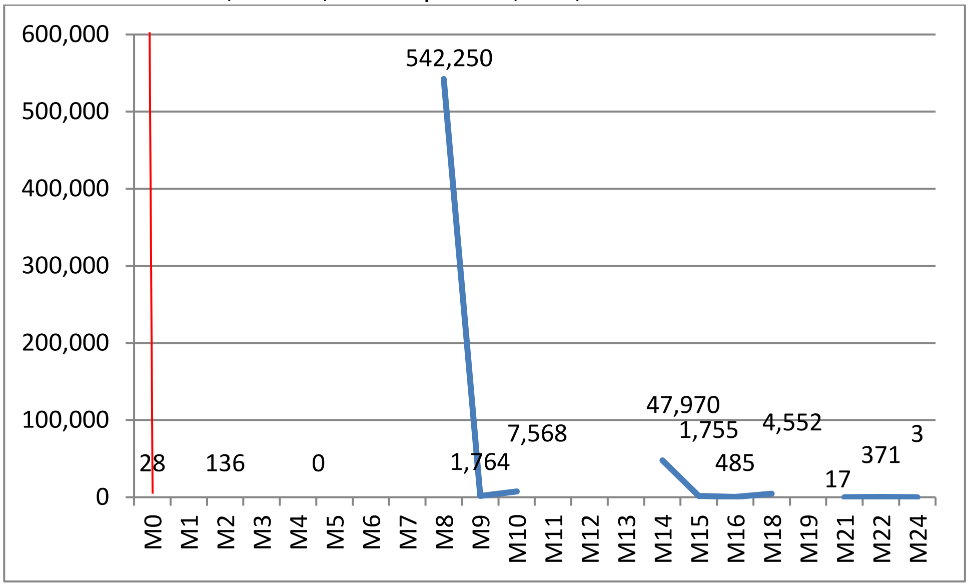


4 VN300131 GIA, MDA 2, Pv 14 episodes, Fx 0, Rx 0

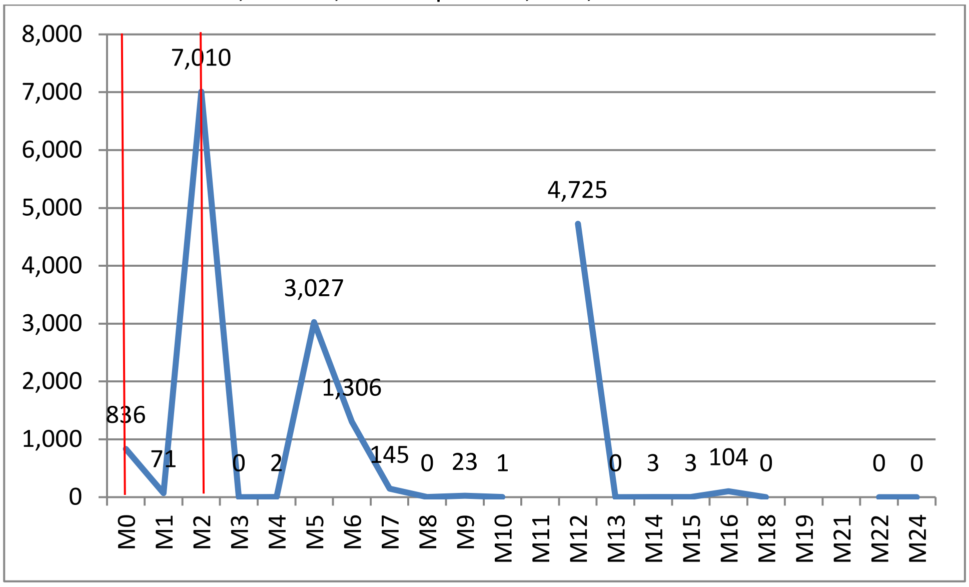


5 VN300438 GIA, MDA 3, Pv 10 episodes, Fx 1, Rx 0

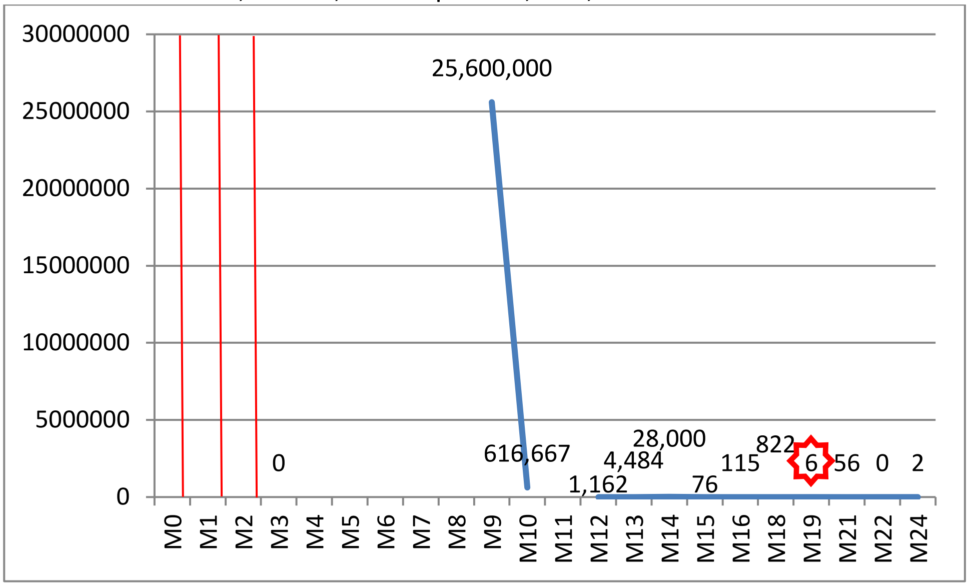

6 VN300462 GIA, MDA 3, Pv 10 episodes, Fx 2, Rx 0

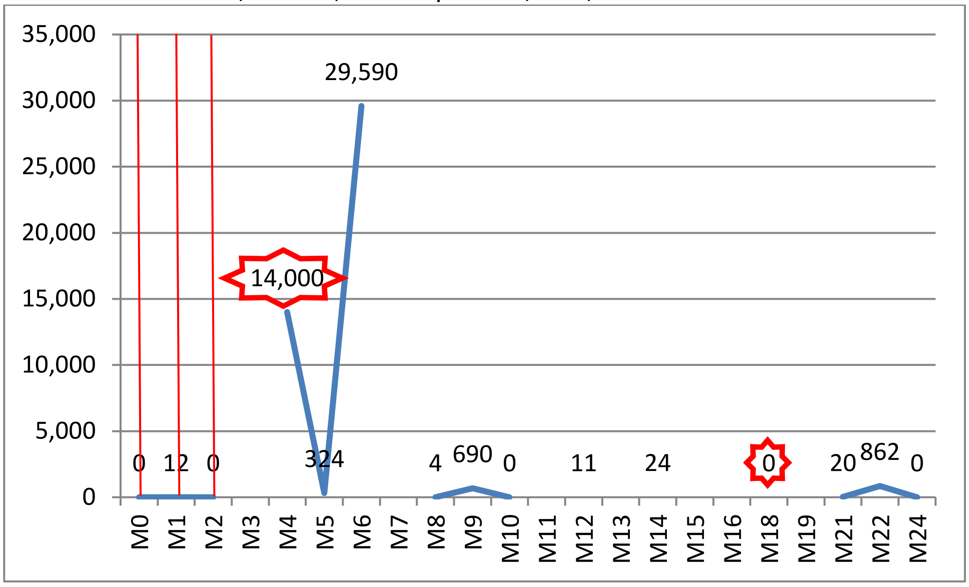


7 VN300532 GIA, MDA 3, Pv 9 episodes, Fx 1, Rx 0

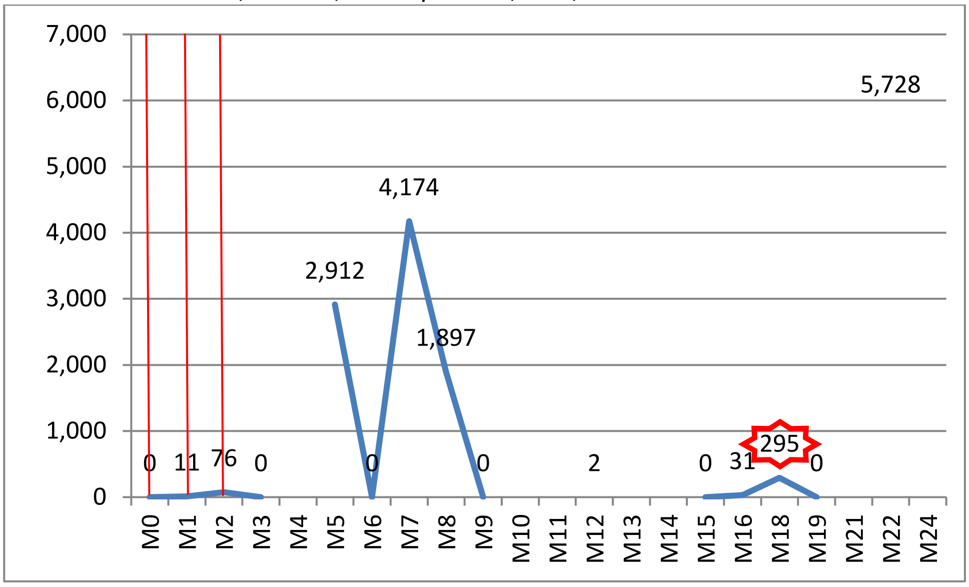


8 VN300128 GIA, MDA 3, Pv 9 episodes, Fx 0, Rx 0

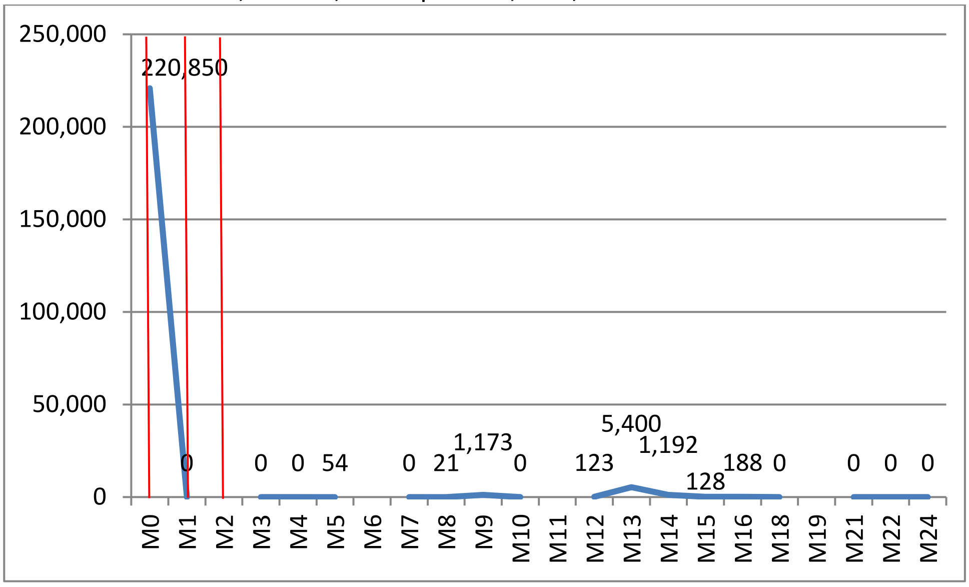

9 VN300418 GIA, MDA 2, Pv 9 episodes, Fx 1, Rx 0

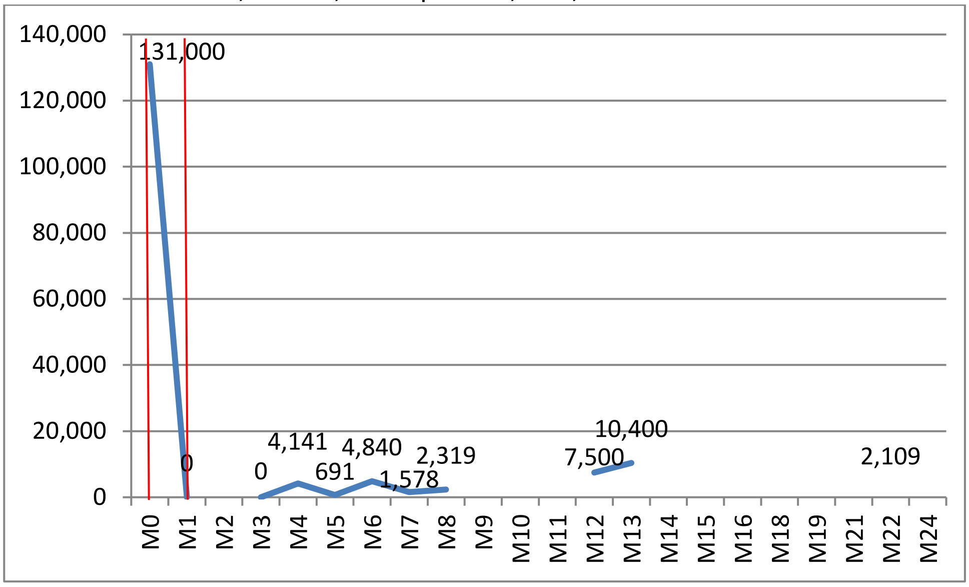


10 VN300403 GIA, MDA 0, Pv 9 episodes, Fx 2, Rx 0

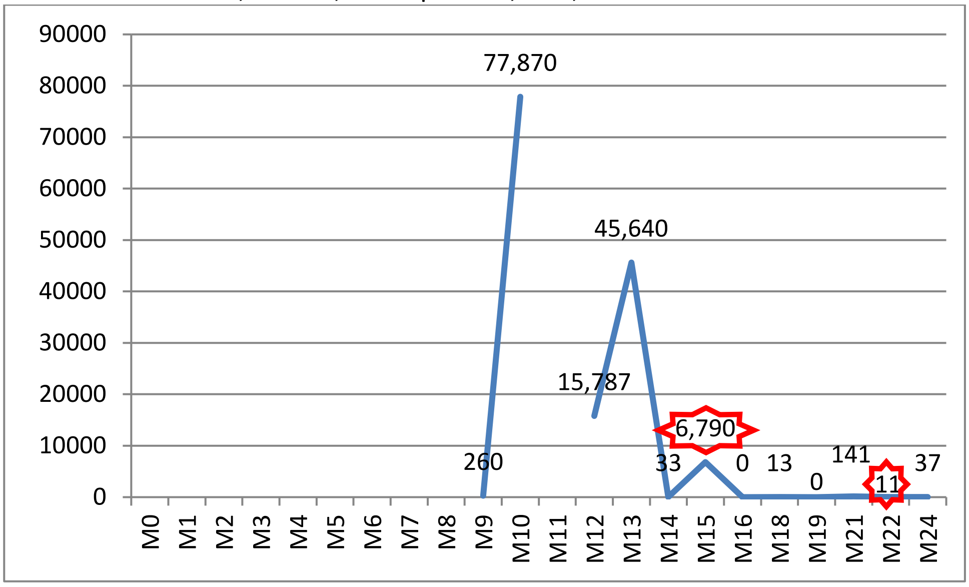


Legend:

GIA = Village
MDA = Mass drug administration
Pf = *Plasmodium falciparum*
Fx = Fever episodes
Rx = Antimalarial treatment received

**Figure S9:** ***Pv, Pf/Pv*** **or** ***P. spp*** **infections - MDA on M12, M13 and M14**

A case series illustrating changes in parasite densities over time in 6 participants with *Pv, Pf/Pv* or *P. spp* infections residing in villages randomised to receive MDA on M12, M13 and M14. The number and timing of MDA(s) received by the participant is indicated by red lines. Parasite densities are provided above the respective month when the sample was collected. A febrile episode is indicated by a red star.

1 VN400249 THA, MDA 0, Pv 10, Fx 0 episodes, Rx 0

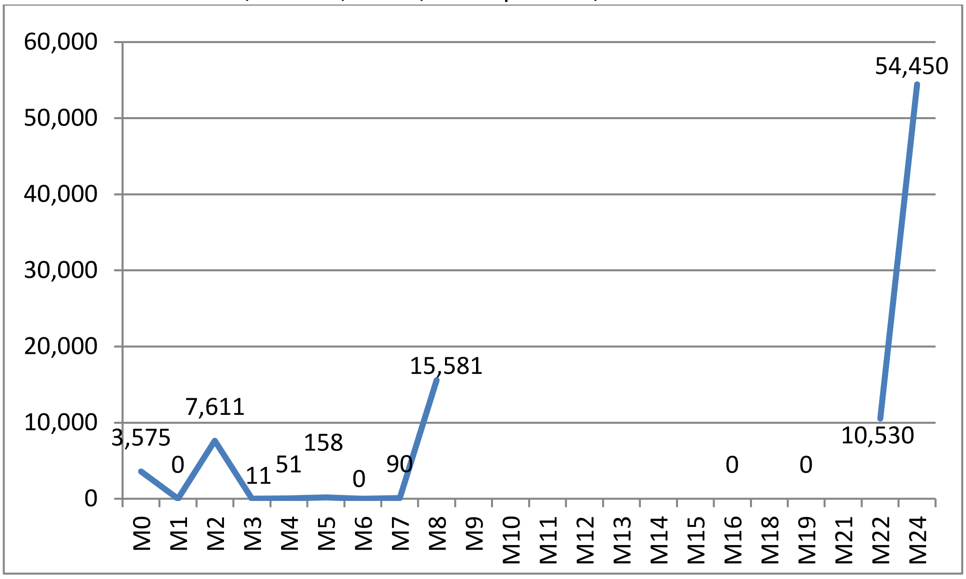


2 VN400380 THA, MDA 2, Pv 10 episodes, Fx 3, Rx 0

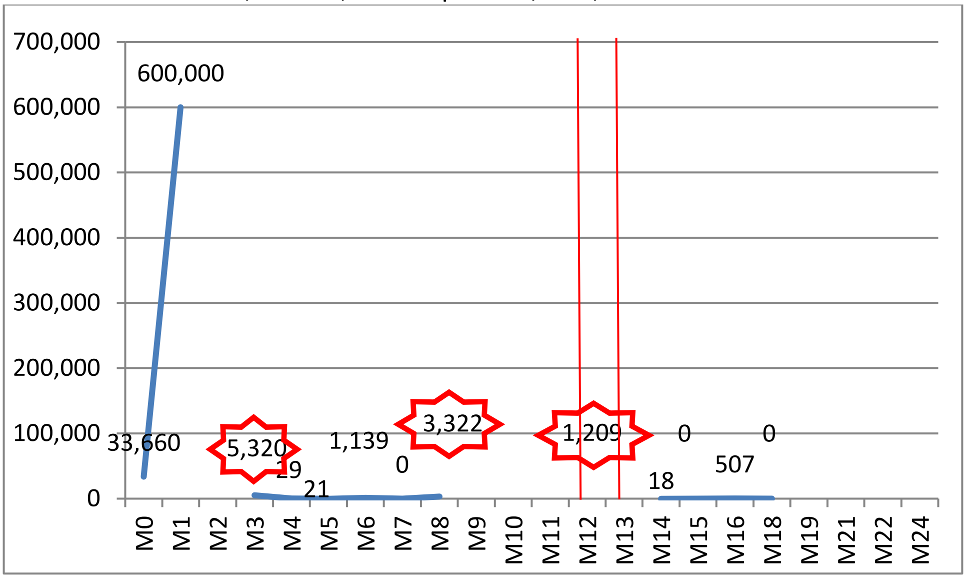

3 VN200827 BB, MDA 2, Pv 8 episodes, Fx 1, Rx 0

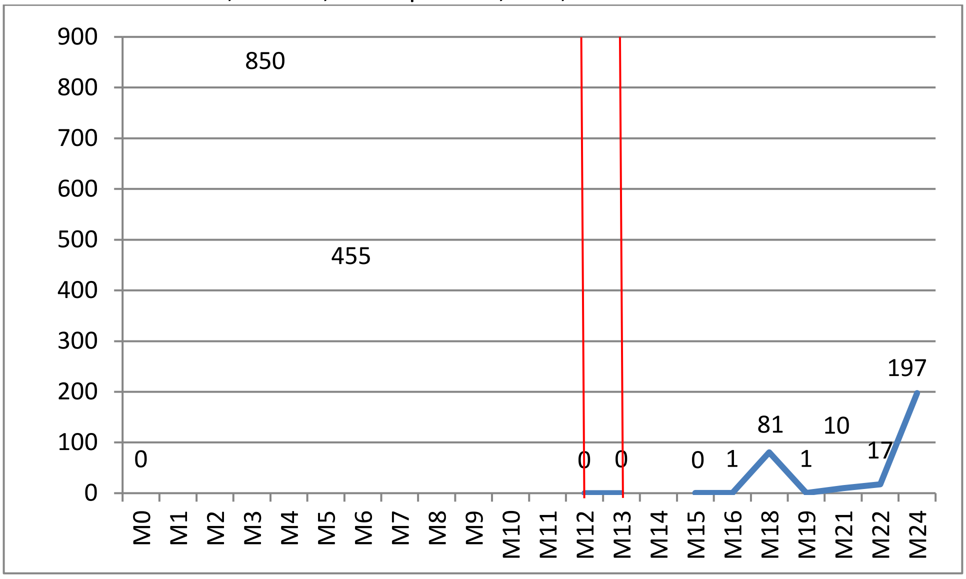


4 VN400405 THA, MDA 3, Pv 8 episodes, Fx 0, Rx 0

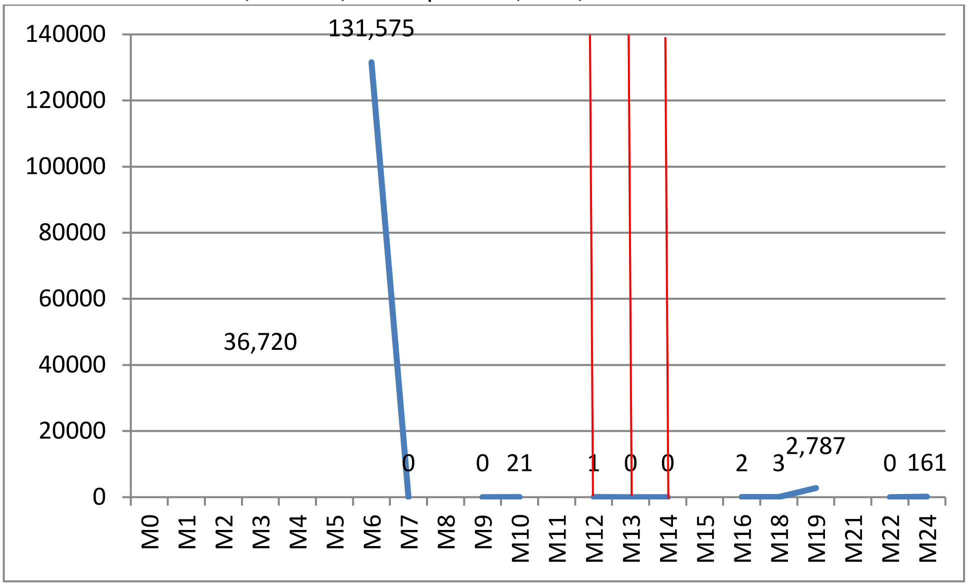


5 VN400379 THA, MDA 2, Pv 8 episodes, Fx 0, Rx 0

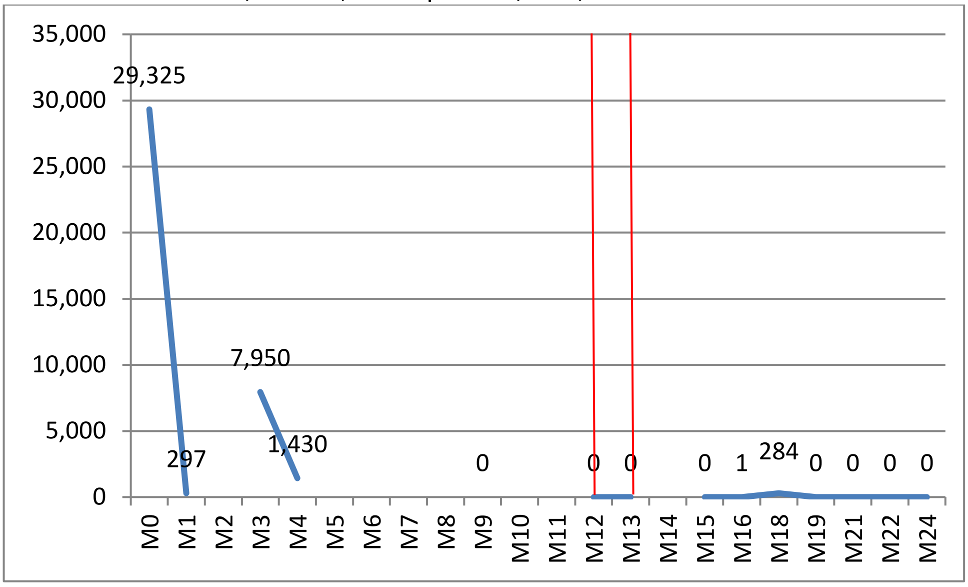

6 VN400093 THA, MDA 1, Pv 8 episodes, Fx 0, Rx 0

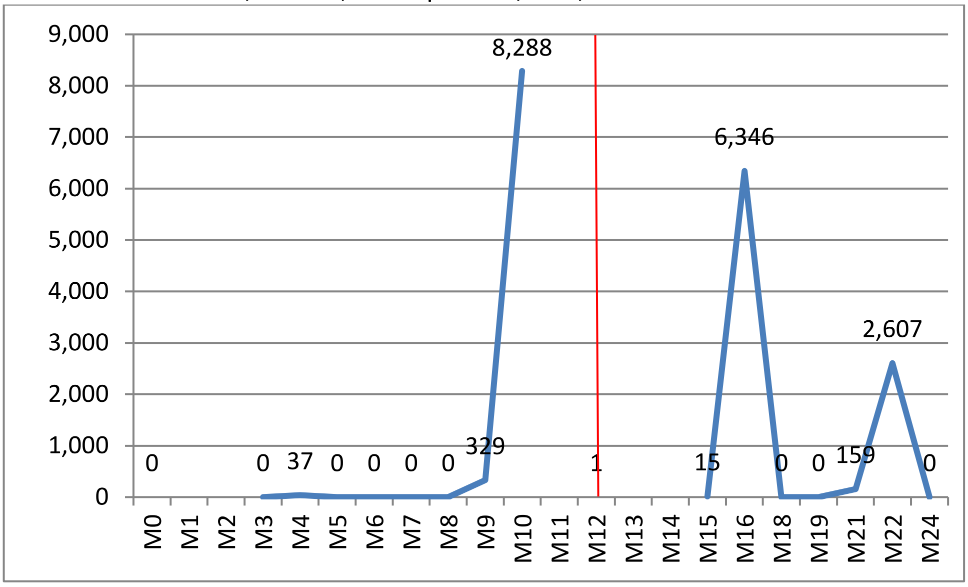


Legend:

THA = Village
MDA = Mass drug administration
Pf = *Plasmodium falciparum*
Fx = Fever episodes
Rx = Antimalarial treatment received
